# Hfq assists small RNAs in binding to the coding sequence of *ompD* mRNA and in rearranging its structure

**DOI:** 10.1261/rna.055251.115

**Published:** 2016-07

**Authors:** Zuzanna Wroblewska, Mikolaj Olejniczak

**Affiliations:** Institute of Molecular Biology and Biotechnology, Faculty of Biology, Adam Mickiewicz University in Poznań, 61-614 Poznań, Poland

**Keywords:** Hfq, sRNA, ribosome, coding sequence, mRNA, *ompD*

## Abstract

The bacterial protein Hfq participates in the regulation of translation by small noncoding RNAs (sRNAs). Several mechanisms have been proposed to explain the role of Hfq in the regulation by sRNAs binding to the 5′-untranslated mRNA regions. However, it remains unknown how Hfq affects those sRNAs that target the coding sequence. Here, the contribution of Hfq to the annealing of three sRNAs, RybB, SdsR, and MicC, to the coding sequence of *Salmonella ompD* mRNA was investigated. Hfq bound to *ompD* mRNA with tight, subnanomolar affinity. Moreover, Hfq strongly accelerated the rates of annealing of RybB and MicC sRNAs to this mRNA, and it also had a small effect on the annealing of SdsR. The experiments using truncated RNAs revealed that the contributions of Hfq to the annealing of each sRNA were individually adjusted depending on the structures of interacting RNAs. In agreement with that, the mRNA structure probing revealed different structural contexts of each sRNA binding site. Additionally, the annealing of RybB and MicC sRNAs induced specific conformational changes in *ompD* mRNA consistent with local unfolding of mRNA secondary structure. Finally, the mutation analysis showed that the long AU-rich sequence in the 5′-untranslated mRNA region served as an Hfq binding site essential for the annealing of sRNAs to the coding sequence. Overall, the data showed that the functional specificity of Hfq in the annealing of each sRNA to the *ompD* mRNA coding sequence was determined by the sequence and structure of the interacting RNAs.

## INTRODUCTION

The Hfq protein is involved in the regulation of translation by bacterial small RNAs (sRNAs) (Updegrove et al. 2016). These noncoding RNAs recognize complementary sequences in their target mRNAs, and induce the activation or repression of translation (Waters and Storz 2009; Updegrove et al. 2015). This regulation is important for the adaptation of enterobacteria to changing environmental conditions (De Lay and Gottesman 2012), maintenance of cellular homeostasis (Papenfort et al. 2013), and virulence of pathogenic species (Papenfort and Vogel 2010; Tree et al. 2014). Hfq is an Sm-like protein, which has a shape of a homohexameric ring (Moller et al. 2002; Zhang et al. 2002). It binds uridine-rich sRNAs using its proximal face (Mikulecky et al. 2004; Sauer and Weichenrieder 2011; Panja et al. 2015) and the outer rim (Sauer et al. 2012), and it binds mRNAs containing (ARN)_*n*_ sequence motifs using its distal face (de Haseth and Uhlenbeck 1980; Link et al. 2009). However, recent studies have suggested that other modes of RNA interactions with Hfq are also possible, both in *Escherichia coli* (Zhang et al. 2013; Małecka et al. 2015; Schu et al. 2015) and in Gram-positive bacteria (Kovach et al. 2014; Robinson et al. 2014).

Hfq participates in sRNA-dependent translation regulation in different ways. The binding of sRNAs by Hfq protects them from degradation (Sledjeski et al. 2001; Moller et al. 2002; Andrade et al. 2012; Fei et al. 2015), but Hfq can also recruit the degradosome to sRNA–mRNA complexes, which leads to their accelerated decay (Ikeda et al. 2011). Moreover, Hfq promotes the pairing of certain sRNAs to their mRNA targets (Moller et al. 2002; Zhang et al. 2002; Maki et al. 2008; Soper and Woodson 2008), and facilitates the annealing of regulatory RNA-OUT to transposase-encoding RNA-IN (Ross et al. 2013). However, other roles of Hfq in translation regulation are also possible. For example, Hfq directly interferes with *sdhC* mRNA translation after being recruited by Spot42 sRNA, which binds to this mRNA independently of Hfq (Desnoyers and Masse 2012). Similarly, the recruitment of Hfq by SgrS sRNA is necessary for *purR* mRNA translation repression (Bobrovskyy and Vanderpool 2016). In another example, Hfq has been shown to compete with RyhB sRNA for binding to *cirA* mRNA, which leads to their opposite functions in the regulation (Salvail et al. 2013). Finally, Hfq has been proposed to repress translation independently of sRNAs by direct binding to *amiE* mRNA (Sonnleitner and Blasi 2014) or to RNA-IN mRNA (Ellis et al. 2015). Overall, these data indicate that Hfq may have different contributions to sRNA stability, their annealing to mRNAs, and translation regulation.

The detailed molecular mechanism used by Hfq has been explained only for its role in the positive regulation of *E. coli rpoS* mRNA by DsrA sRNA, which targets the 5′-untranslated region of this mRNA (Soper and Woodson 2008; Soper et al. 2011; Peng et al. 2014a,b). Hfq forms multilateral interactions with *rpoS* mRNA, resulting in a distorted, more compact structure of mRNA, which facilitates the annealing of DsrA to *rpoS* (Soper et al. 2011; Peng et al. 2014a). The binding of Hfq to an (ARN)_4_ sequence motif in *rpoS* mRNA, which correctly positions Hfq in relation to the DsrA binding site*,* is essential for the DsrA–*rpoS* pairing (Soper and Woodson 2008; Peng et al. 2014b). However, it is not known whether a similar mechanism is used by Hfq to contribute to translation regulation by other sRNAs, especially those which bind to the mRNA coding sequence and exert negative regulation of translation.

Recent Hfq profiling data in a pathogenic *E. coli* strain showed that almost 40% of recovered reads mapped to the coding regions (Tree et al. 2014), which suggested that the coding sequence could be an important target of Hfq-dependent regulation. Indeed, although the majority of sRNAs target the 5′-untranslated regions, several *Salmonella* and *E. coli* sRNAs bind within the coding sequence of mRNAs (Bouvier et al. 2008; Pfeiffer et al. 2009; Gutierrez et al. 2013; Papenfort et al. 2013; Guo et al. 2014; Bobrovskyy and Vanderpool 2016). Some sRNAs bind just downstream from the start codon and are expected to interfere with the initiation of translation (Bouvier et al. 2008; Balbontin et al. 2010; Papenfort et al. 2010; Guo et al. 2014). The annealing sites of other sRNAs are located downstream from the footprint of the initiation complex (Pfeiffer et al. 2009; Frohlich et al. 2012; Gutierrez et al. 2013; Bobrovskyy and Vanderpool 2016), despite the fact that the elongating ribosome is expected to efficiently unwind helical structures on its path (Takyar et al. 2005; Qu et al. 2011). The coding sequence of *Salmonella ompD* mRNA contains the binding sites of four sRNAs, which are RybB (Papenfort et al. 2010), SdsR (Frohlich et al. 2012), InvR (Pfeiffer et al. 2007), and MicC (Pfeiffer et al. 2009). Among them RybB sRNA represses the initiation of translation (Papenfort et al. 2010), while MicC induces the accelerated decay of *ompD* mRNA, which is dependent on RNase E (Pfeiffer et al. 2009; Bandyra et al. 2012). The coimmunoprecipitation studies showed that *ompD* mRNA was bound by Hfq (Sittka et al. 2008). Moreover, the OmpD protein expression was increased in *hfq* deletion strains, which suggested the involvement of Hfq in *ompD* translation regulation (Sittka et al. 2007; Bossi et al. 2008). However, it is not known how Hfq participates in this regulation.

In order to evaluate the role of Hfq in the sRNA-dependent regulation of the *Salmonella ompD* mRNA, the kinetics of annealing of three sRNAs, RybB, SdsR, and MicC, to the coding region of *ompD* mRNA were compared. Moreover, the truncated variants of these sRNAs were used to dissect the contributions of Hfq to the formation of each individual pair. Finally, the RNA structure probing was used to map the secondary structure of *ompD* mRNA and to monitor the conformational changes induced by the sRNA annealing.

## RESULTS

**FIGURE 1.**
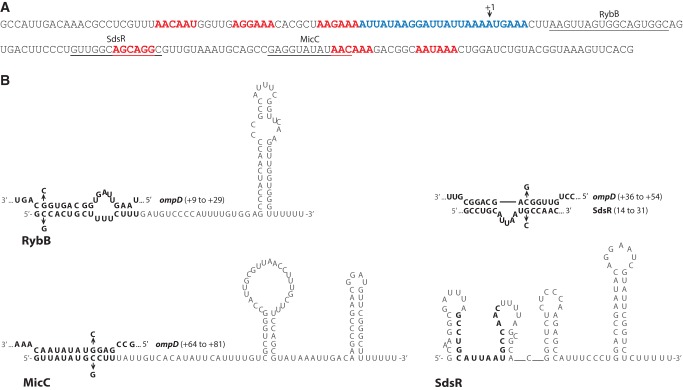
The leader region of *ompD* mRNA and the secondary structures of sRNAs RybB, SdsR, and MicC. (*A*) The sequence of the *ompD*-187 mRNA. The RybB, SdsR, and MicC sRNA binding sites are underlined. (ARN)_2_ motifs are marked with red font, and the AU-rich region with blue font. (*B*) Secondary structures of sRNAs RybB (Bouvier et al. 2008), MicC (Pfeiffer et al. 2009), and SdsR (Supplemental Fig. S1), with pairing to complementary *ompD* mRNA sequences shown on the structures of RybB and MicC, and *above* the structure of SdsR. The pairing region of SdsR is marked on its structure with bold font. The location of complementary regions within the *ompD* coding sequence is shown in brackets. The mutations used to confirm the sRNA–mRNA interactions (Supplemental Fig. S2) are shown at the structures of complementary regions.

The analysis of the sequence of *ompD* mRNA showed that its 5′-terminal region containing the binding sites of RybB, SdsR, and MicC sRNAs includes several sequence motifs that could serve as Hfq binding sites (Fig. 1). Among them are six (ARN)_2_ repeats (named below as ARN-1 to ARN-6) located in the 5′-UTR and in the coding sequence. One of those motifs (termed ARN-3) is followed by a long AU-rich region located mainly in the 5′-UTR (Fig. 1A). Both ARN repeats and AU-rich sequences can serve as Hfq binding sites in mRNA molecules. For example, an (ARN)_4_ motif is important for Hfq binding and DsrA sRNA annealing to *rpoS* mRNA (Soper and Woodson 2008), an AU-rich sequence is involved in the regulation of *chiP* mRNA, while both ARN repeats and AU-rich regions serve in the regulation of *csgD* mRNA by different sRNAs (Schu et al. 2015). Hence, these data suggest that the *ompD* mRNA leader sequence could contain potential Hfq binding sites.

### Hfq binds tightly to the *ompD* mRNA

**FIGURE 2.**
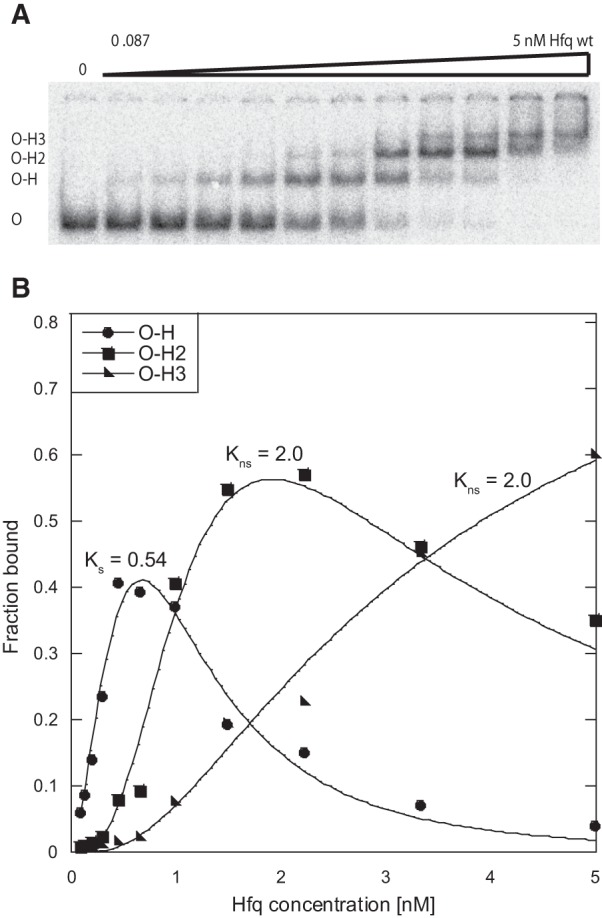
The equilibrium binding of Hfq to the *ompD* mRNA leader. (*A*) The analysis of Hfq binding to ^32^P-labeled *ompD*-187 using the native mobility shift assay. The *ompD*-187 complexes with Hfq are marked as O–H, O–H2, and O–H3. (*B*) The plot of ^32^P-*ompD*-187 binding data from *A* versus the concentration of Hfq. The data were fit to a partition function assuming one specific and two equal nonspecific binding sites. The equilibrium dissociation constant values obtained by fitting the data are shown on the plot, and the average values are presented in Table 1.

To determine the affinity of Hfq to the *ompD* mRNA, a native gel mobility shift assay was used (Table 1; Fig. 2). In these experiments the 187-nt long 5′-terminal fragment of *ompD* mRNA (*ompD*-187) was used, which contains the binding sites of RybB, SdsR, and MicC sRNAs (Fig. 1). This *ompD* fragment was sufficient for the in vivo regulation of translation by MicC sRNA, which binds the deepest of them in the coding sequence (Pfeiffer et al. 2009). The data showed that Hfq formed three complexes with *ompD*-187 at a range of Hfq concentrations up to 5 nM (Fig. 2A). The formation of higher order complexes with Hfq has also been previously observed for other RNAs, for example, for *E. coli rpoS* mRNA (Soper and Woodson 2008), RNA-IN mRNA (Ross et al. 2013), and DsrA sRNA (Lease and Woodson 2004). Hfq bound *ompD* mRNA leader very tightly with the equilibrium dissociation constant (*K*_d_) value of 0.61 ± 0.17 nM for the tightest complex (per hexamer). This was similar to the *K*_d_ value of 0.24 nM reported for the 160-nt long fragment of RNA-IN mRNA (Ross et al. 2013), and tighter than the 50 nM value reported for the 301-nt long fragment of *rpoS* mRNA (Peng et al. 2014b) or the 30 nM value reported for the 182-nt long fragment of *glmS* mRNA (Salim et al. 2012). These results demonstrated the direct binding of Hfq to *ompD* mRNA (Fig. 2), which was suggested by Hfq coimmunoprecipitation studies (Sittka et al. 2008) and by Hfq binding to the binary complexes of ^32^P-labeled RybB with unlabeled *ompD* (Papenfort et al. 2006). Moreover, these results suggested a specific interaction of Hfq with *ompD* mRNA, because Hfq bound this mRNA with subnanomolar affinity (Table 1) comparable to those of other mRNAs, for which the functional role of Hfq binding was previously determined (Ross et al. 2013; Peng et al. 2014a).

**TABLE 1.**
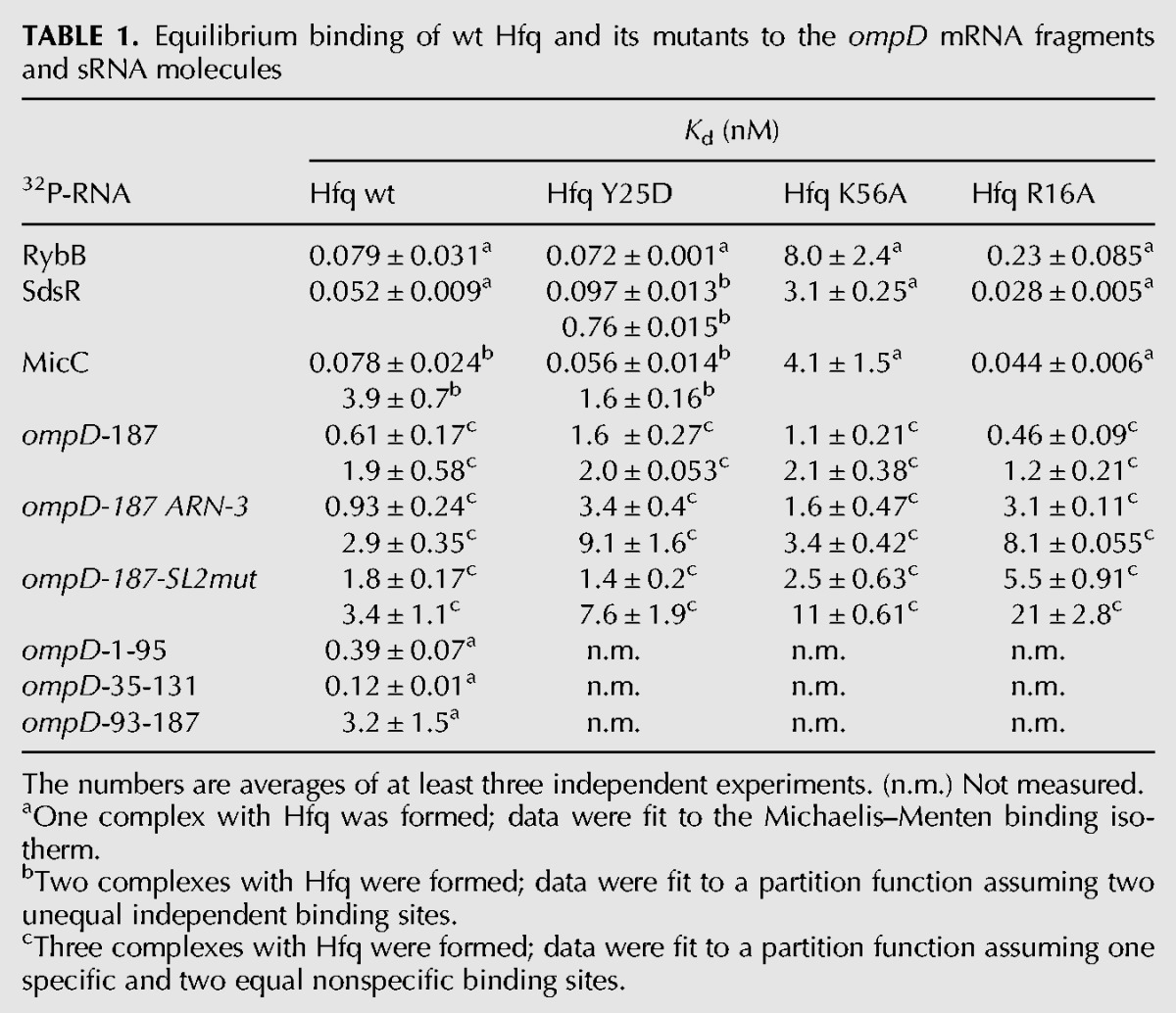
Equilibrium binding of wt Hfq and its mutants to the *ompD* mRNA fragments and sRNA molecules

To better understand how Hfq interacts with *ompD* mRNA, the binding of *ompD*-187 to wild-type (wt) Hfq and its variants with mutations in the distal (Y25D), proximal (K56A), or rim (R16A) surface of the ring was compared (Table 1). The *K*_d_ value for the tightest complex of *ompD*-187 with Hfq Y25D was threefold weaker than with wt Hfq. At the same time, the *K*_d_ value for the tightest complex with Hfq K56A was twofold weaker, and with Hfq R16A was not changed as compared to the wild-type protein (Table 1). The modest effect of the Y25D mutation on the distal face indicated that other RNA binding sites on Hfq were involved, and partly compensated for the loss of the thermodynamic contribution of the distal surface contacts. The simultaneous use of several sites on Hfq was also observed for its binding to *fhlA* mRNA (Salim and Feig 2010), where both the distal and the proximal surfaces were used, and to *rpoS* mRNA (Peng et al. 2014a) and *csgD* mRNA (Schu et al. 2015), where both the distal and the rim surfaces were involved. These data suggest that the involvement of several RNA binding sites on the Hfq ring is a general feature of Hfq interactions with mRNA molecules.

The binding of Hfq to sRNAs RybB, SdsR, and MicC was mainly dependent on the proximal face contacts (Table 1). These sRNAs bound to wt Hfq with very tight affinities similar to those determined before for *E. coli* sRNAs (Olejniczak 2011). The Y25D mutation in the distal face of Hfq did not affect their binding, while the K56A mutation in the proximal face resulted in up to 100-fold weaker binding. The R16A mutation on the rim of Hfq showed a small effect on the binding of RybB, but not of SdsR or MicC. The use of the proximal surface for binding to Hfq is characteristic of Class I sRNAs (Zhang et al. 2013; Schu et al. 2015), which are directed to this site by their 3′-terminal oligouridine sequences remaining from Rho-independent terminators (Sauer and Weichenrieder 2011). Consistently, the sRNAs studied here do not contain ARN repeats (Fig. 1B), which direct other sRNAs to the distal face of Hfq (Olejniczak 2011; Małecka et al. 2015; Schu et al. 2015). Overall, these data suggest that Hfq could use the proximal surface of its ring to recruit RybB, SdsR, and MicC sRNAs toward the *ompD* mRNA coding sequence.

### Hfq accelerates the annealing of RybB, SdsR, and MicC sRNAs to the *ompD* mRNA

**FIGURE 3.**
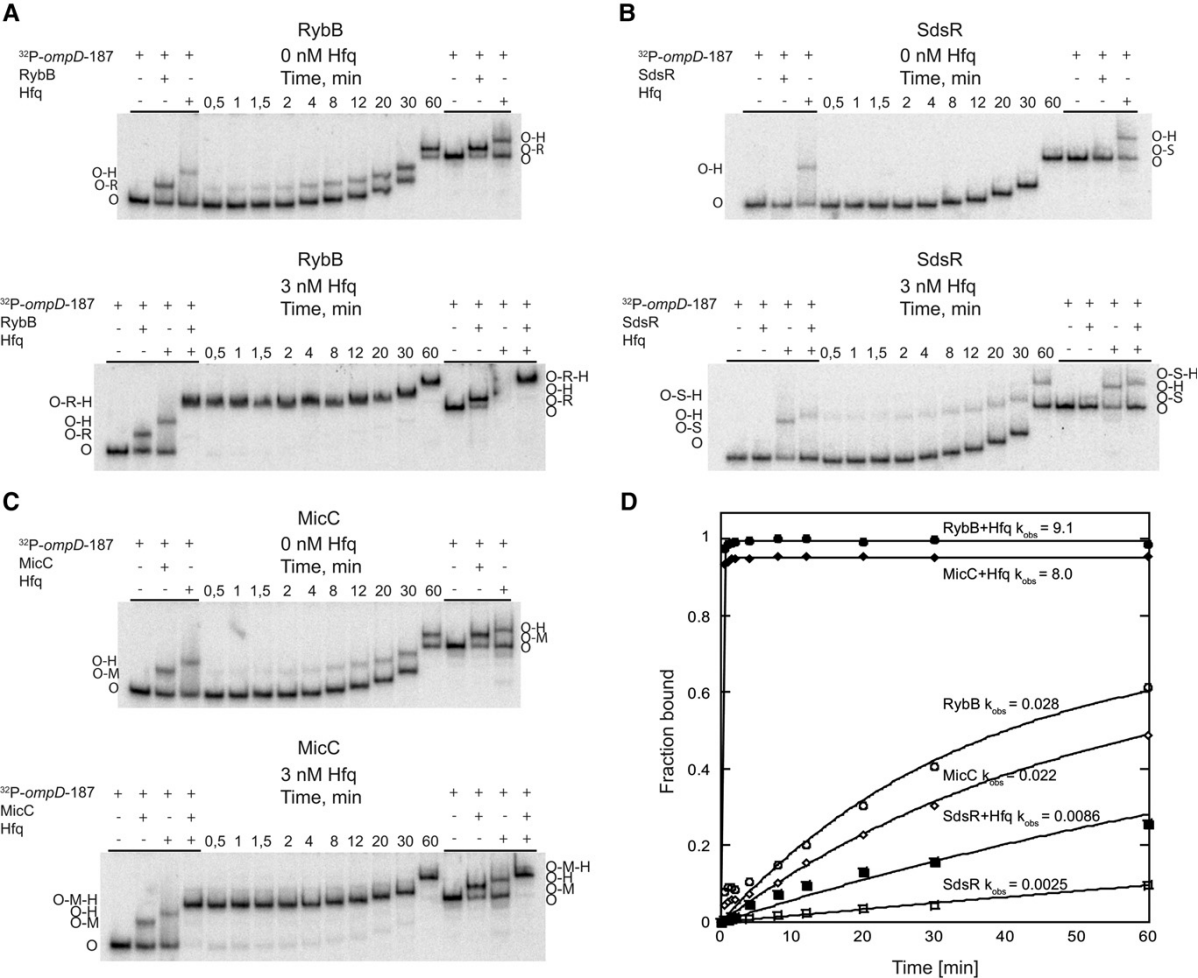
Hfq accelerates the association of RybB, SdsR, and MicC sRNAs to *ompD* mRNA. The kinetics of annealing of ^32^P-labeled *ompD*-187 (1 nM concentration) to (*A*) RybB, (*B*) SdsR, or (*C*) MicC sRNA at 25 nM concentration, in the absence or presence of 3 nM Hfq. Free *ompD*-187 is marked as O, *ompD*-187-Hfq complex as O-H, *ompD*-187-RybB complex as O-R, *ompD*-187-RybB-Hfq ternary complex as O-R-H, *ompD*-187-SdsR complex as O-S, *ompD*-187-SdsR-Hfq ternary complex as O-S-H, *ompD*-187-MicC complex as O-M, and *ompD*-187-MicC-Hfq ternary complex as O-M-H. The control reactions, in which ^32^P-*ompD*-187 alone was bound to Hfq, were supplemented with 2 nM cold *ompD*-187 mRNA to obtain the total concentration of RNA equal to that of Hfq. (*D*) The data from *A*, *B*, and *C* were plotted versus time, and the fitting of data provided *k*_obs_ values, which are presented on the plot. The average *k*_obs_ values are shown in Table 2.

To elucidate the role of Hfq for the annealing of sRNAs to the coding sequence of *ompD* mRNA, the kinetics of association of RybB, SdsR, and MicC sRNAs to the 5′-^32^P-labeled *ompD*-187 mRNA were analyzed (Fig. 3). The progress of annealing reactions was monitored using a native gel mobility shift assay in electrophoretic buffer containing 2 mM Mg^2+^ as previously described (Peng et al. 2014b). The rates of association (*k*_obs_) were measured at 1 nM concentration of ^32^P-labeled *ompD*-187 and 25 nM unlabeled sRNA in the presence or absence of 3 nM Hfq. For each of the three studied sRNAs, the control reactions showed distinct differences in the electrophoretic mobility of free *ompD*-187, its binary complexes with Hfq or sRNA, and the ternary complex of *ompD*-187 with Hfq and sRNA.

The rate of RybB sRNA annealing to the *ompD* mRNA leader was strongly accelerated by Hfq (Table 2; Fig. 3A,D). In the absence of Hfq, the rate of RybB sRNA annealing to ^32^P-labeled *ompD*-187 was 0.032 min^−1^. However, when 3 nM Hfq was present, a ternary complex was formed rapidly with a rate that was more than 250-fold faster (Table 2; Fig. 3A,D). This effect was comparable to that for DsrA annealing to a 301-nt fragment of *rpoS* mRNA, where Hfq induced 60-fold faster association (Peng et al. 2014b). To test whether base-pairing between RybB and *ompD*-187 was required to form the ternary complex with Hfq, the effect of mutations in the complementary regions of RybB (mutation C2G) and *ompD*-187 (mutation G94C) was studied (Fig. 1B). These mutations were detrimental for translation regulation in vivo (Bouvier et al. 2008). Indeed, the RybB C2G mutant failed to form a complex with *ompD*-187 mRNA (Supplemental Fig. S2A), and the annealing of RybB to the *ompD*-187 G94C mutant was weakened in comparison to the natural *ompD*-187 sequence (Supplemental Fig. S2B). However, the annealing of RybB C2G mutant to *ompD*-187 G94C mutant was restored to the wild-type level (Supplemental Fig. S2C). This confirms that in the ternary complex RybB is directly paired to *ompD*-187, as opposed to binding independently to different sites on Hfq.

**TABLE 2.**
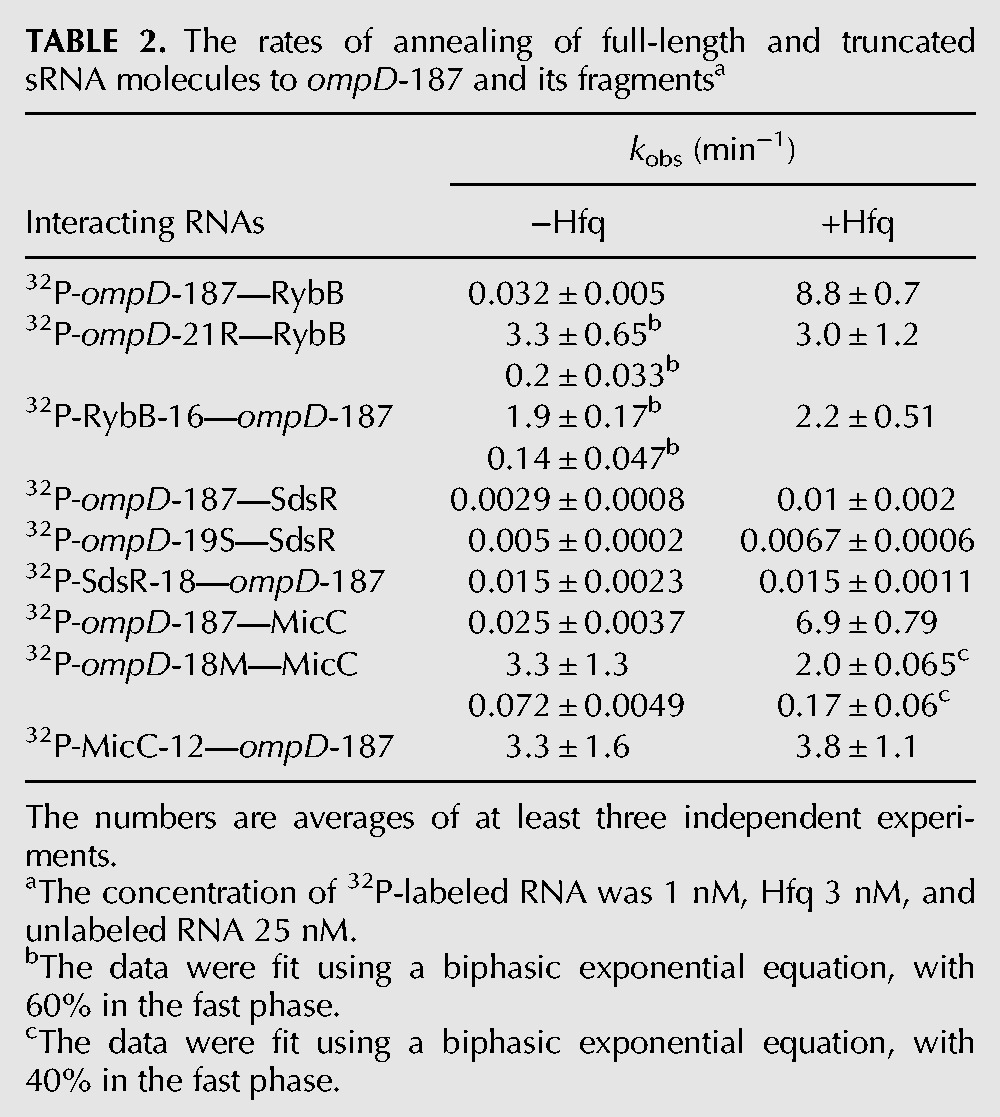
The rates of annealing of full-length and truncated sRNA molecules to *ompD*-187 and its fragments^a^

The annealing of SdsR sRNA to the *ompD* mRNA leader in the presence of Hfq proceeded much more slowly than that of RybB (Table 2; Fig. 3B,D). In the absence of Hfq, SdsR sRNA annealed to *ompD*-187 with a rate of 0.0029 min^−1^. In the presence of Hfq, a ternary complex was formed with only a threefold faster rate (Table 2). The mutations in complementary positions in SdsR (the G26C mutation) and in *ompD*-187 (the C113G mutation) were detrimental for annealing when tested individually (Supplemental Fig. S2D,E), in agreement with their effect on translation regulation (Frohlich et al. 2012). Consistently, the annealing was restored to the wild-type level when molecules with compensatory mutations were studied (Supplemental Fig. S2F).

Hfq also had a strong influence on the rates of MicC sRNA annealing to *ompD* (Table 2; Fig. 3C,D). In the absence of Hfq, the rate of MicC annealing to ^32^P-labeled *ompD*-187 was 0.025 min^−1^. In the presence of Hfq, the annealing rate was more than 250-fold faster, which was comparable to the effect observed for RybB annealing (Table 2). The mutations in complementary positions in MicC (the C9G mutation) and in *ompD*-187 (the G139C mutation) were detrimental for annealing when tested individually (Supplemental Fig. S2G,H), in agreement with their effect on *ompD* regulation in vivo (Pfeiffer et al. 2009). The MicC sRNA annealing to mRNA was restored, when molecules with compensatory mutations were used (Supplemental Fig. S2I). Overall, these data showed that the annealing rates of all three sRNAs were increased by Hfq, although the effects were ranging from as little as threefold for SdsR to more than 200-fold for RybB and MicC.

To elucidate the involvement of Hfq binding sites, the annealing of RybB, SdsR, and MicC sRNAs to *ompD*-187 was measured in the presence of Hfq variants with mutations in the sites of RNA binding (Table 3). The data showed that both the mutation Y25D in the distal face and the mutation K56A in the proximal face resulted in more than 100-fold slower annealing of RybB to ompD-187, while the rim mutation had a small effect. The annealing of MicC was also affected by both the distal and the proximal face mutations, which had about a 25-fold effect, while the rim mutation had a fivefold effect (Table 3). As the annealing of SdsR in the presence of wt Hfq was already slow, the Hfq mutations in the proximal and distal face had only about a twofold effect, and the rim mutation did not affect the annealing. Because the equilibrium binding data showed absolute preference of sRNAs RybB, SdsR, and MicC to bind to the proximal face of Hfq (Table 1), this suggested that *ompD* mRNA binds to the distal face of Hfq, in agreement with the detrimental effect of the Y25D mutation on the annealing (Table 3).

**TABLE 3.**
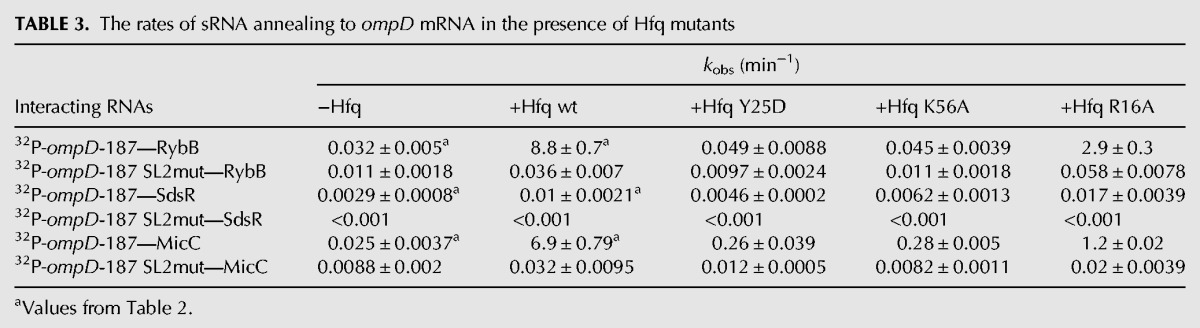
The rates of sRNA annealing to *ompD* mRNA in the presence of Hfq mutants

### Hfq differently contributes to the annealing of sRNAs RybB, SdsR, and MicC to *ompD* mRNA

The role of Hfq in the annealing of each sRNA to *ompD* mRNA was further dissected using minimal sRNA molecules and complementary *ompD* mRNA fragments (Table 2; Fig. 1B; Supplemental Fig. S3). The study of the annealing of truncated variants of interacting RNAs should allow us to evaluate the contributions of their structures to the energetic barrier preventing their annealing in the absence of Hfq. A similar approach has been used previously to reveal that the essential role of Hfq in DsrA sRNA annealing to *rpoS* mRNA was to rearrange the *rpoS* mRNA structure (Soper et al. 2011). The minimal sRNA molecules consisting of the sequence that is complementary to *ompD* (shown in bold in Fig. 1B) were RybB-16, which was effective in repression of *ompN* mRNA translation (Bouvier et al. 2008), SdsR-18, and MicC-12. The minimal fragments of *ompD* mRNA corresponding to the binding sites of these sRNAs were *ompD*-21R, *ompD*-19S, and *ompD*-18M, respectively. None of the six short RNAs bound Hfq at the concentrations used in the annealing assays (Supplemental Fig. S3).

The data suggested that the structures of both RybB sRNA and *ompD* mRNA contributed to the energetic barrier preventing their efficient annealing (Table 2; Supplemental Fig. S3). In the absence of Hfq, both the truncated RybB-16 fragment and the truncated *ompD*-21R fragment bound to *ompD*-187 and RybB, respectively, much faster than full-length RybB to *ompD*-187 (Table 2; Supplemental Fig. S3A,B,G,H). In the presence of Hfq, both truncated fragments bound to the full-length molecules faster than in its absence, with rates that were comparable to that of full-length RybB annealing to *ompD*-187 (Table 2). These results suggested that the role of Hfq in the annealing of RybB to *ompD*-187 was to rearrange the structures of both interacting RNAs.

The contribution of Hfq to the annealing of SdsR sRNA to *ompD*-187 depended on the structures of both interacting molecules, but the effects of the RNA truncation were relatively modest (Table 2; Supplemental Fig. S3C,D,G,H). In the absence of Hfq, the rates of SdsR-18 annealing to *ompD*-187 were fivefold faster, and those of *ompD*-19S annealing to SdsR were twofold faster than for the annealing of SdsR to *ompD*-187. This suggested that the role of Hfq in the full-length SdsR annealing to *ompD*-187 was to rearrange the structures of both interacting RNAs. However, Hfq did not further increase the rates of annealing of these RNA pairs.

In contrast, the structure of MicC sRNA was the main barrier to its annealing to *ompD*-187 (Table 2; Supplemental Fig. S3E,F,G,H). Both in the absence and presence of Hfq the rates of annealing of minimal MicC-12 to *ompD*-187 were similar to the rates of full-length MicC annealing to *ompD*-187 in the presence of Hfq. This suggested that in the context of the structure of *ompD*-187, the MicC binding site was easily accessible for MicC-12. In contrast, both in the absence and presence of Hfq the truncated *ompD*-18M bound to full-length MicC much slower than *ompD*-187 did. *ompD*-18M had biphasic rates of annealing to MicC with a faster rate responsible for 40% of the binding (Table 2; Supplemental Fig. S3). Overall, these data suggested that the structure of MicC could be an important barrier to its annealing to *ompD* mRNA, and thus would necessitate the use of Hfq for unfolding. Alternatively, a role of Hfq could be to optimally position the pairing sequence of the full-length MicC toward its binding site in *ompD* mRNA. Regardless of the detailed explanation, these data suggested that the contribution of Hfq to MicC sRNA annealing was mostly determined by the structure of MicC, and not by the structure of *ompD* mRNA.

### The *ompD* mRNA leader sequence folds into five stem–loops with RybB, SdsR, and MicC binding sites located in different structural contexts

**FIGURE 4.**
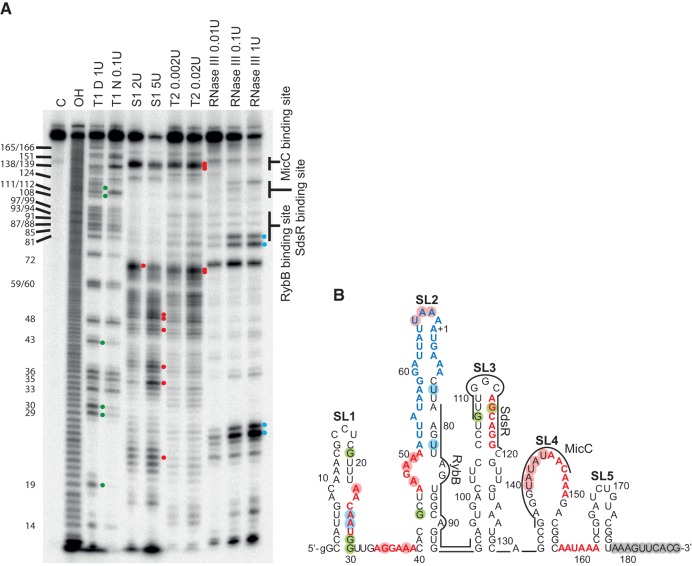
The binding sites of RybB, SdsR, and MicC sRNAs have different structural contexts within the *ompD* mRNA leader. (*A*) The structure probing of 5′-^32^P-labeled *ompD*-187 using RNases indicated *above* the lanes. The untreated *ompD*-187 sample was resolved in lane C, formamide ladder in lane OH, and the products of reactions with RNase T1 in denaturing or native conditions in lanes marked T1 D and T1 N, respectively. The positions of G-specific cleavages by RNase T1 are indicated on the *left* side of the gel. (*B*) The secondary structure model of *ompD*-187 proposed by the RNAstructure program with hard constraints (circled positions) obtained from structure probing experiments in *A*. Residues constrained as single-stranded are indicated with red circles, while double-stranded are indicated with blue circles (RNase III) or green circles (RNase T1 in native conditions). Gray circles indicate nucleotides constrained as single-stranded based on experiments comparing the cleavage patterns of *ompD*-187 and 3′-end-truncated *ompD*-177 (data not shown). The AU-rich region is marked in blue, and (ARN)_2_ motifs in red. The binding sites of RybB, SdsR, and MicC sRNAs are indicated by lines.

To elucidate how the structural context of sRNA binding sites could affect their annealing, the in vitro structure probing of the *ompD*-187 mRNA was performed using structure-specific ribonucleases (Fig. 4). Nuclease S1 and RNase T2 were used to identify structurally dynamic regions, while the location of the double-stranded regions was inferred from the comparison of RNase T1 induced cleavages in denaturing and native conditions, and from degradation induced by RNase III. The 3′-terminal 10-nt sequence of *ompD*-187 was predicted to be single-stranded, because the 3′-truncated 10-nt shorter derivative of *ompD* leader had the same pattern of cleavages as *ompD*-187 (data not shown). The probing data were used as constraints to predict the *ompD* mRNA structure using RNAstructure software (Reuter and Mathews 2010). The data showed that the 5′-terminal region of *ompD* mRNA was organized into five stem–loop structures, named below as SL1 to SL5 (Fig. 4B). The cleavage patterns of three shorter mRNA fragments containing the sequences corresponding to stem–loops SL1 and SL2 (*ompD*-1-95), SL2 and SL3 (*ompD*-35-131), or SL3, SL4, and SL5 (*ompD*-93-187), were the same as the patterns of the corresponding regions in the 187-nt-long *ompD* mRNA leader (data not shown), which confirmed that the sequences involved in the predicted five stem–loop structures indeed formed separate structure modules.

The probing data showed that each of the three sRNA binding sites was located in a different structural context in *ompD* mRNA (Fig. 4). The binding site of RybB sRNA was located in a structurally dynamic SL2 motif, the binding site of SdsR in a stable apical region of SL3, and the binding site of MicC in an extended loop of SL4 (Fig. 4B). The 5′-terminal sequence and the apical loop of SL2, including the ARN-3 motif and the AU-rich region, were susceptible to cleavage by single-strand specific nucleases, which suggested that this region was quite dynamic. The RybB binding site was adjacent to the ARN-3 motif and the AU-rich region because it was located in the complementary strand of SL2. On the other hand, the binding site of SdsR sRNA, and the overlapping ARN-4 motif, were located in the apical portion of the SL3 motif (Fig. 4B). This element of *ompD* mRNA secondary structure was likely quite stable, because there were few cleavages with any used nucleases in this region, except for the apical triloop of SL3 (Fig. 4A).

The unique feature of the MicC sRNA binding site was its location in the extended purine-rich 16-nt apical loop of SL4, the stem of which was stabilized by four GC pairs (Fig. 4). The loop of SL4 was defined by a series of strong cleavages by RNase T2 and nuclease S1 at residues 140–144. The absence of cleavages at 145–150 could indicate the presence of noncanonical interactions within the loop. The single-stranded character of this region was further supported by degradations induced in native conditions by RNase T1 at G138 and G139 at the 5′ end of this loop, and at residue G151 at its 3′ end. The presence of the stable stem of SL4 was supported by the decreased T1 cleavage in native conditions at residue G133. The ARN-5 motif, which partly overlaps the MicC binding site, was located in the loop of SL4, and the nearby ARN-6 motif was located in the single-stranded region between SL4 and SL5.

### The annealing of sRNAs RybB and MicC induces rearrangements in the structure of the *ompD* mRNA leader

**FIGURE 5.**
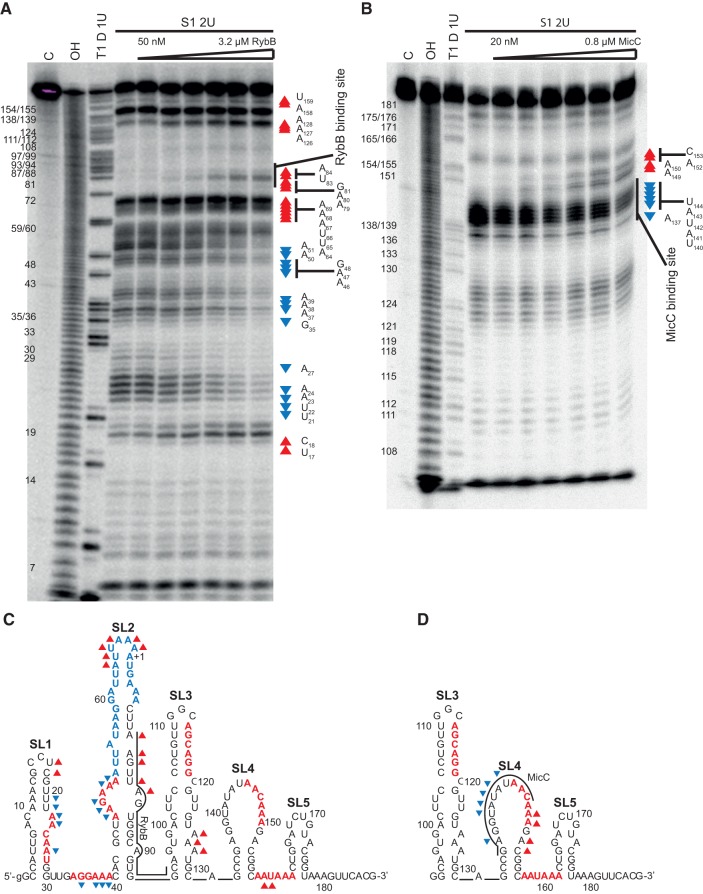
The binding of RybB and MicC sRNAs induces local conformational changes in the *ompD* mRNA leader. (*A*) The structure probing of 5′-^32^P-labeled *ompD*-187 with nuclease S1 at increasing concentrations of RybB sRNA. (*B*) The structure probing of 5′-^32^P-labeled *ompD*-93–187 with nuclease S1 at increasing concentrations of MicC sRNA. The untreated samples were resolved in lanes marked C, formamide ladders in lanes OH, and reactions with RNase T1 in denaturing conditions in lanes marked T1 D. The positions of G-specific cleavages by RNase T1 are indicated on the *left* sides of the gels. The changes in nucleotide susceptibility to cleavage upon RybB or MicC binding are shown on the secondary structure of *ompD*-187 in *C*, and *ompD*-93-187 in *D*, respectively. The nucleotide positions, which showed higher susceptibility to degradation by nuclease S1 in the presence of respective sRNA, are marked by red triangles, and those which showed lower susceptibility are marked by blue reverse triangles.

The annealing of RybB sRNA to *ompD*-187 induced extensive changes in the cleavage pattern of stem–loops SL1 and SL2, and the intervening sequence (Fig. 5A,C). While the degradation of apical portions of SL1 and SL2 increased with the concentration of RybB, the linker between SL1 and SL2 together with the adjacent regions became more protected (Fig. 5A,C). The increased cleavages at positions 79–81 and 83–84 (Fig. 5A,C) marked the 5′ edge of the RybB binding site in mRNA. It was previously reported that the 5′ terminal sequence of RybB was complementary to two partly overlapping duplicated sequences in *ompD* mRNA, with the downstream sequence considered as the more likely binding site (Balbontin et al. 2010; Papenfort et al. 2010). The increased cleavages observed here are consistent with RybB binding to the downstream binding site, which confirms that conclusion. In contrast to the extensive conformational rearrangements induced by RybB, the structure-probing pattern of *ompD*-187 was not changed in the presence of SdsR sRNA (Supplemental Fig. S4). The lack of SdsR-dependent changes in the cleavage pattern suggests that the binding of SdsR protects this region from cleavage or that bound SdsR causes only limited unfolding of *ompD* mRNA structure.

The binding of MicC sRNA induced specific local changes in the probing pattern of the 3′ part of the SL4 loop and the adjacent stem (Fig. 5B,D). The nuclease S1 probing of the *ompD* mRNA fragment including the nucleotides from 93 to 187 (*ompD*-93-187) showed that the binding of MicC sRNA protected the apical loop nucleotides 137 and 140–144 in SL4, which are within the MicC binding site. Interestingly, it also stimulated the cleavage of residues 149, 150, 152, and 153 (Fig. 5B), which are located immediately 3′ of the MicC binding site. The cytosine 153 is involved in the top base pair of the SL4 stem, which would be unfolded by MicC pairing to the sequence including complementary G136. In contrast, the adenosines 149, 150, and 152 are part of the sequence of five purines in the SL4 loop, which are not predicted to form canonical Watson–Crick interactions. However, large loops are often stabilized by noncanonical bonding patterns (Halder and Bhattacharyya 2013), and a continuous purine sequence is also expected to be stabilized by stacking interactions. The disruption of these intra-loop interactions by MicC binding would explain the increased susceptibility of this region to degradation by nuclease S1. Interestingly, the adenosine 152 (position 83 of the coding sequence) was also reported as a site of increased RNase E cleavage of *ompD* mRNA upon MicC binding in vivo (Pfeiffer et al. 2009). Hence, the data suggest that the annealing of MicC increases the conformational dynamics of the mRNA region 3′ adjacent to its binding site, in agreement with the increased susceptibility of this region to RNase E cleavage upon MicC binding in vivo.

### The Hfq binding site in the 5′-untranslated region of *ompD* mRNA is important for sRNA annealing to the coding sequence

**FIGURE 6.**
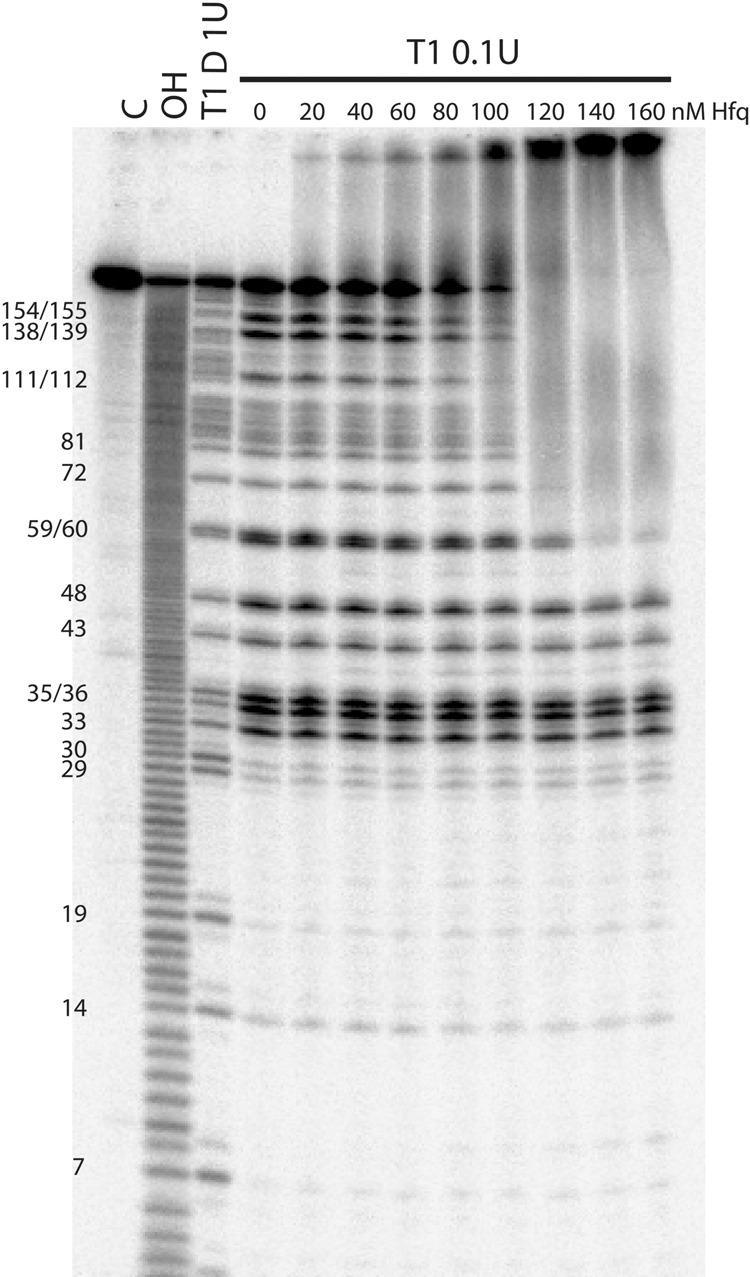
The boundary experiments with 5′-^32^P-labeled *ompD*-187 mRNA suggest that it contains a strong Hfq binding site in the 5′-untranslated region. The denaturing gel analysis of the binding of Hfq to the 5′-labeled *ompD*-187 degradation fragments obtained by partial digestion with RNase T1. The untreated *ompD*-187 sample was resolved in the lane marked C, the formamide ladder in lane OH, and reaction with RNase T1 in denaturing conditions in lane T1 D. The positions of G-specific cleavages by RNase T1 are indicated on the *left* side of the gel.

To identify the location of Hfq binding sites in *ompD* mRNA, a boundary binding assay was used (Fig. 6; Supplemental Fig. S5). This assay took advantage of the fact that Hfq remains tightly bound to its RNA ligands even in the presence of 8 M urea (Brescia et al. 2003). After 5′-^32^P-labeled *ompD*-187 was partly degraded with RNase T1 (Fig. 6) or nuclease S1 (Supplemental Fig. S5A), the reaction products were incubated with Hfq. This was followed by separation of Hfq-bound from unbound RNA fragments by denaturing gel electrophoresis (Fig. 6; Supplemental Fig. S5). At Hfq concentrations above 100 nM, only the 5′-terminal RNA fragments of at least 60 nt in length were shifted by Hfq on a denaturing gel. This suggested the presence of a strong Hfq binding site in the 3′ part of this 60-nt long region, which coincides with the location of the sequence composed of ARN-3 motif followed by the AU-rich sequence. When the boundary experiment was performed using the 3′-end labeled *ompD*-187, the minimal fragments bound included the region from about position 110–187, which contained motifs ARN-5 and ARN-6 (Supplemental Fig. S5B,C). Overall, these data suggested that *ompD*-187 contains at least two Hfq binding sites, one of which is located in the untranslated region and the other in the coding sequence of this mRNA.

To investigate the relative strengths of these two potential Hfq binding sites, the affinities of Hfq for three overlapping fragments of *ompD* mRNA were determined (Table 1). The *ompD*-1-95 fragment contained regions SL1 and SL2, *ompD*-35-131 contained SL2 and SL3, and *ompD*-93-187 contained SL3, SL4, and SL5 (Fig. 4B). The data showed that the Hfq affinity for *ompD*-1-95 was similar to that for *ompD*-187, while the affinity for *ompD*-35-131 was fourfold tighter. Both of these constructs contained SL2, which included the ARN-3 motif followed by the AU-rich region. In contrast, the Hfq binding affinity of *ompD*-93-187, which contained motifs ARN-5 and ARN-6, was sixfold weaker than that of *ompD*-187. These data are consistent with the presence of an important Hfq binding site in SL2, which is present in both *ompD*-1-95 and *ompD*-35-187 mRNA fragments, but not in *ompD*-93-187.

To test whether ARN sequence repeats present at several regions of *ompD* mRNA participate in Hfq binding and sRNA annealing, the effect of point mutations in the (ARN)_2_ motifs was analyzed (Supplemental Fig. S6; Supplemental Tables S1, S2). The secondary structure of *ompD*-187 mutants with substitutions in the repeats ARN-1, ARN-2, ARN-3, ARN-5, and ARN-6 was conserved, as predicted by RNAstructure software (Supplemental Fig. S6). The ARN-4 motif was not mutated, because it is located in a stable secondary structure region. The equilibrium binding data showed that none of the mutations affected the stability of the tightest complex of *ompD*-187 with Hfq (Supplemental Table S1). Only a modest, less than threefold decrease in the stability of the weaker complexes with Hfq was observed when both ARN-5 and ARN-6 were mutated. It is consistent with the observation that mutations in (AAN)_4_ motif in *rpoS* mRNA had a detrimental effect on the formation of higher order complexes with Hfq (Peng et al. 2014b). When the rates of RybB annealing to *ompD*-187 mutants were measured, all of the mutants had a modest, less than threefold, detrimental effect on the annealing (Supplemental Table S2). The rates of MicC annealing to *ompD*-187 with ARN-1, ARN-2, or ARN-3 mutations were similarly affected, while the mutations of ARN-5 and ARN-6 had much larger effects. However, because these mutations were adjacent to the MicC binding site, they could have affected its annealing directly and not via their influence on the Hfq binding. Moreover, it was previously proposed that the binding of sRNA and Hfq to the same site in mRNA is mutually exclusive (Beisel et al. 2012), and the MicC binding site overlaps with part of the ARN-5 motif (Fig. 1A). Hence, these results were not conclusive about the role of ARN sequences in Hfq binding and sRNA annealing to *ompD* mRNA.

**FIGURE 7.**
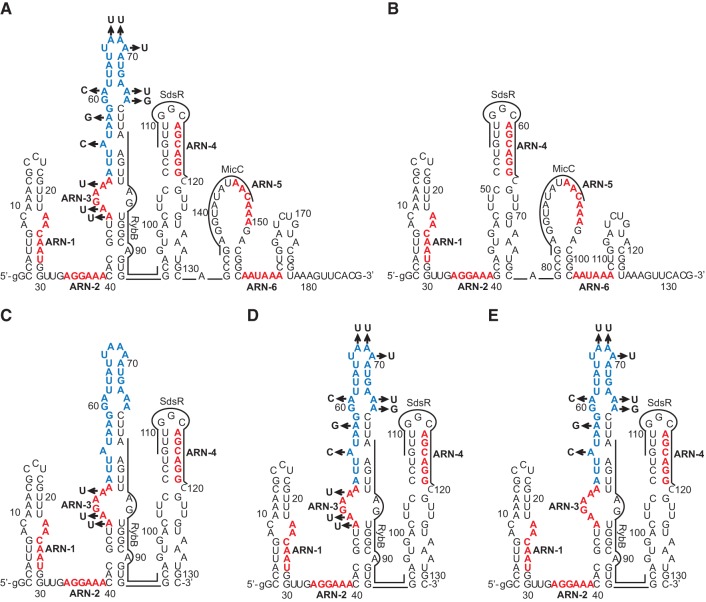
The nucleotide substitutions in SL2 region presented on the structure of *ompD*-187 or *ompD*-131. Point mutations used to generate (*A*) *ompD*-187-SL2mut, (*B*) *ompD*-187-ΔSL2, (*C*) *ompD*-131 ARN3, (*D*) *ompD*-131-SL2mut, (*E*) *ompD*-131-AU mRNA mutants. The (ARN)_2_ motifs and the AU-rich sequence are marked in red and blue, respectively.

To elucidate the importance of the SL2 region as a functional Hfq binding site, several adenosine residues in the ARN-3 motif and the following AU-rich sequence were substituted with other nucleotides, thus creating the *ompD*-187-SL2mut construct (Table 4; Fig. 7A). The data showed that the rate of RybB annealing to this mRNA mutant was 200-fold slower than to the wt *ompD*-187, and 100-fold slower than to the *ompD*-187-ARN-3 mutant (Table 4). Importantly, Hfq protein induced about a 200-fold increase in the rate of RybB annealing to wt *ompD*-187 as compared to only threefold for the *ompD*-187-SL2mut. This suggested the crucial importance of the AU-rich region for the RybB annealing. To confirm this conclusion, a shorter *ompD*-131 construct, containing 5′ terminal 131 nt, and its variants *ompD*-131-SL2mut, *ompD*-131 ARN-3, and *ompD*-131-AU were used (Fig. 7C,D,E). The rate of RybB annealing to *ompD*-131-SL2mut, which contained the same substitutions as *ompD*-187-SL2mut, was 50-fold slower than to wt *ompD*-131 and 30-fold slower than to *ompD*-131 ARN-3 (Table 4). However, when only the AU-rich region was mutated (*ompD*-131-AU), the rate was similar to that for the *ompD*-131-SL2mut construct. This confirmed the important role of the AU-rich region for the Hfq-dependent annealing of RybB to *ompD* mRNA. Further analysis showed that the annealing of MicC and SdsR sRNAs was also detrimentally affected by the mutations in the *ompD*-187-SL2mut construct (Table 4). The rate of MicC annealing to *ompD*-187-SL2mut was 200-fold slower than to wt *ompD*-187, and 70-fold slower than to *ompD*-187 ARN-3, while the rates of SdsR annealing to *ompD*-187-SL2mut were too slow to be accurately measured. The removal of the whole SL2 region in the *ompD*-187-ΔSL2 construct also negatively affected the MicC annealing but its effect was smaller than that of the mutations in *ompD*-187-SL2mut (Table 4; Fig. 7B). Overall, these data suggested that the long AU-rich sequence in the 5′-untranslated region of *ompD* is important for Hfq-dependent annealing of all three sRNAs to the coding sequence of this mRNA.

**TABLE 4.**
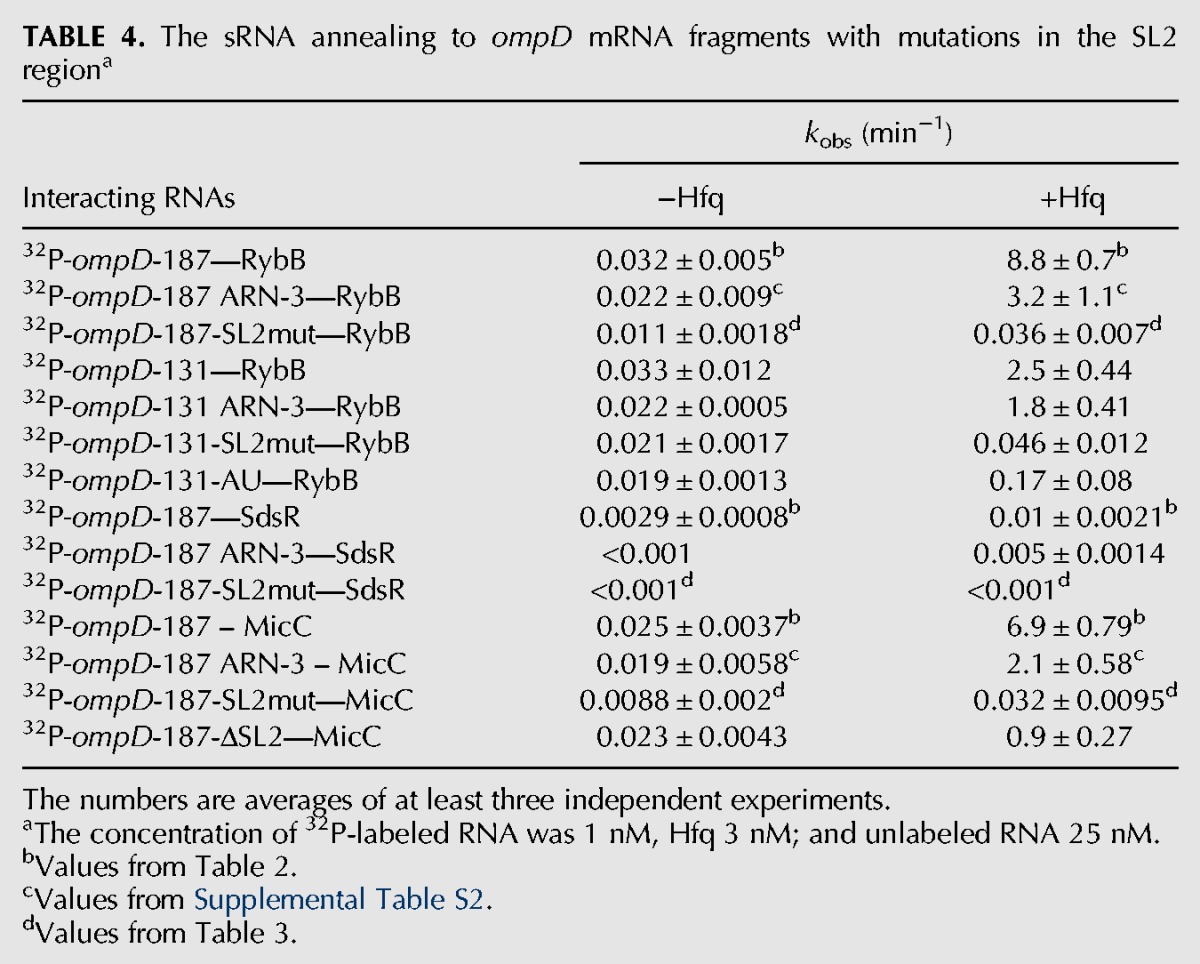
The sRNA annealing to *ompD* mRNA fragments with mutations in the SL2 region^a^

To better understand how Hfq interacts with the SL2 region, the equilibrium binding of Hfq mutants to the *ompD*-187-SL2mut construct and their role in the sRNA annealing to this mRNA were analyzed (Tables 1, 3). The affinity of the *ompD*-187-SL2mut construct to Hfq was about threefold weaker than that of wt *ompD*-187, and about twofold weaker than that of *ompD*-187 ARN-3, which contained only the mutations in the ARN-3 motif (Table 1). The affinity of Hfq with mutations Y25D and K56A to *ompD*-187-SL2mut or *ompD*-187 ARN-3 was similar to that of wt *ompD*-187. However, the R16A Hfq mutant bound sixfold weaker to *ompD*-187 ARN-3, and 10-fold weaker to *ompD*-187-SL2mut than to wt *ompD*-187. This suggests that contacts with the nonspecific rim binding site of Hfq compensate for the lost interactions with the distal face of Hfq in mRNAs that contain mutations in the ARN-3 motif and the AU-rich region. To further investigate how Hfq interacts with the AU-rich region, the rates of RybB annealing to *ompD*-187-SL2mut were compared in the presence of the Y25D, K56A, and R16A mutants of Hfq (Table 3). Even though RybB annealing to *ompD*-187-SL2mut was much slower than to *ompD*-187, the proximal and distal face mutations had the same detrimental role in RybB annealing, while the rim site did not affect it. The same mutations also had similar effects on the MicC annealing to *ompD*-187-SL2mut, while the annealing of SdsR to this mRNA construct was too slow to accurately measure (Table 3). This suggested that the residual Hfq-dependent annealing of RybB and MicC sRNAs to *ompD*-187-SL2mut depended on the remaining contacts with the distal face of Hfq, and not on the alternative interactions with Hfq. Overall, these data indicated the essential role of the Hfq distal face contacts with the long AU-rich sequence in *ompD* mRNA for the sRNA annealing.

## DISCUSSION

The binding of Hfq to the 5′-untranslated region of *ompD* mRNA allows it to increase the rates of annealing of RybB, SdsR, and MicC sRNAs to the coding sequence (Table 2; Fig. 3). The Hfq-dependent acceleration of sRNA annealing to the mRNA untranslated region has been previously observed, for example, for OxyS annealing to *fhlA* mRNA (Zhang et al. 2002; Salim and Feig 2010), Spot42 to *galK* mRNA (Moller et al. 2002), and RNA-OUT to RNA-IN (Ross et al. 2013). However, the contribution of Hfq has been explored in molecular detail only for DsrA sRNA annealing to the 5′-untranslated region of *rpoS* mRNA (Soper and Woodson 2008; Soper et al. 2011; Panja and Woodson 2012; Peng et al. 2014a,b). Hfq had about a 60-fold effect on the *k*_obs_ rate of DsrA annealing to *rpoS* mRNA (Peng et al. 2014b), and about a 20-fold on the rate of RNA-OUT annealing to RNA-IN (Ross et al. 2013). The data presented here showed that Hfq increased the rates of RybB and MicC annealing to the coding sequence of *ompD* mRNA about 250-fold, and it also had a small effect on the rate of SdsR annealing (Table 2). Hence, similarly to those sRNAs, which bind to the untranslated regions, also sRNAs that bind to the coding sequence can use Hfq to increase their rates of annealing to the target mRNAs.

The Hfq protein contributed differently to the annealing of each of the three sRNAs to *ompD* mRNA (Table 2; Supplemental Fig. S3). The role of Hfq in the annealing of RybB to *ompD* mRNA was to overcome the energetic barriers formed by the structures of both interacting RNAs (Table 2). Hence, it was similar to the Hfq contribution to the annealing of DsrA sRNA, which involved mainly the rearrangement of *rpoS* mRNA structure, but was also partly dependent on the structure of DsrA (Soper et al. 2011). The contribution of Hfq to the annealing of SdsR was also dependent on the structures of both SdsR sRNA and *ompD* (Table 2; Supplemental Fig. S3). However, the Hfq-dependent increase of the SdsR annealing rate was much lower than for the other two sRNAs. In contrast, the structure of MicC sRNA appeared to be the crucial barrier to its annealing to *ompD* (Table 2; Supplemental Fig. S3). The minimal MicC sRNA in the absence of Hfq bound to *ompD* mRNA as fast as the full-length MicC in the presence of Hfq. This indicated that the main contribution of Hfq to the MicC annealing involved the rearrangement of the MicC structure or the recruitment of full length MicC towards its binding site in mRNA. These data suggested that the contributions of Hfq to each sRNA annealing to mRNA are individually adjusted depending on the structures of the interacting RNAs.

The long AU-rich sequence in the 5′-UTR served as the functionally important Hfq binding site in *ompD* mRNA (Tables 3, 4; Figs. 6, 7). This sequence consists of an (AAN)_2_ repeat, which was named here as ARN-3, followed by a 24-nt long region, which contains adenosines at each third position making it an (ANN)_8_ motif (Fig. 1). Overall, this whole 30-nt long sequence contains 18 adenosines and eight uridines. The Hfq binding sites identified previously in mRNAs included both the ARN repeats (Soper and Woodson 2008; Link et al. 2009; Beisel et al. 2012) and the AU-rich regions (Schu et al. 2015; Updegrove et al. 2016). However, it was recently proposed that there is a continuum of possible interactions of Hfq with different RNA sequences (Schu et al. 2015). Consistently, it was also reported that Hfq binding sites in *E. coli* transcriptome often contained mismatches in ARN repeats (Tree et al. 2014). The dependence of RybB, SdsR, and MicC binding on the proximal face of Hfq (Table 1) is typical for Class I sRNAs (Zhang et al. 2013; Schu et al. 2015). This suggests that Hfq could use its unoccupied distal surface to bind *ompD* mRNA, when matching these sRNAs to their binding sites in *ompD* mRNA. Indeed, the annealing of RybB and MicC sRNAs to *ompD* was strongly detrimentally affected by the mutations in the proximal and distal face of Hfq, while the effect of the mutation in the rim was smaller (Table 3). In agreement with that, the location of adenosines in each third position of the AU-rich region is consistent with the distance between the adenosine specific binding pockets on the distal surface of Hfq (Link et al. 2009). Regardless of the detailed mechanism of Hfq binding to the AU-rich region of *ompD* mRNA, these data support the view that Hfq can use different binding modes to bring together the pairing regions of mRNA and sRNA molecules.

The rearrangements of mRNA structure are an important part of translation regulation mechanisms dependent on sRNAs. The Hfq protein rearranges the structure of *rpoS* mRNA to facilitate the annealing of DsrA sRNA (Soper et al. 2011), and the structure of *sodB* mRNA to promote the annealing of RyhB sRNA (Geissmann and Touati 2004). The mRNA structure rearrangements can also be induced by sRNAs. This is most evident for the three sRNAs that bind to the 5′-UTR of *rpoS* mRNA and change the equilibrium between ribosome-accessible and inaccessible conformations of this mRNA (Soper et al. 2010). The data presented here showed that the sRNA binding can also induce conformational changes in the *ompD* mRNA (Fig. 5). The binding of RybB sRNA induced rearrangements within 5′-UTR and at the beginning of the *ompD* coding sequence (Fig. 5A,C), while MicC induced local rearrangements in the coding sequence, which were consistent with MicC pairing to the large 16-nt loop of SL4 and the unfolding of the stem of SL4 (Fig. 5B,D). The sequence directly 3′ adjacent to the MicC binding site, which unfolding was detected by increased nuclease S1 cleavage (Fig. 5B), coincides with the region of increased RNase E cleavage in vivo (Pfeiffer et al. 2009), which may reflect the increased structural dynamics of this region upon MicC annealing. In another recent example, the conformational change induced by the annealing of MicF sRNA to the coding sequence of *lpxR* mRNA resulted in increased susceptibility to cleavage by RNase E (Corcoran et al. 2012). A different outcome followed the binding of SR1 sRNA to the coding sequence of *ahrC* mRNA of *B. subtilis*, which induced changes in the mRNA structure resulting in the repression of the initiation of translation (Heidrich et al. 2007).

Hfq promotes the annealing of sRNAs, which use different mechanisms in the regulation of *ompD* mRNA translation in vivo. Among the three sRNAs studied here, RybB binds at the beginning of the *ompD* coding sequence within the five-codon window, and interferes with the initiation of translation (Bouvier et al. 2008; Balbontin et al. 2010; Papenfort et al. 2010). SdsR anneals to *ompD* mRNA outside of the footprint of the initiating ribosome and is expected to affect mRNA decay rather than translation initiation (Frohlich et al. 2012). In contrast, the mode of action of MicC was proposed to involve the recruitment of RNase E to its binding site in mRNA leading to the accelerated decay of *ompD* mRNA (Pfeiffer et al. 2009; Bandyra et al. 2012). The fact that Hfq facilitates the annealing of sRNAs acting differently on translation (Table 2; Fig. 3) suggests that it has a general role in promoting the sRNA–mRNA pairing, regardless of the downstream effects of sRNA binding. Indeed, the same Hfq binding site in the 5′-UTR of *ompD* mRNA is used to promote the annealing of sRNAs RybB and MicC, which have different locations within the coding sequence and different mechanisms of action. However, it is also possible that the binding of Hfq to the downstream site within the coding sequence of *ompD*, which is not essential for sRNA annealing, could play distinct roles, such as the recruitment of RNase E, in this way affecting the regulation.

In summary, the data presented here show that a long AU-rich sequence in the 5′-untranslated region of the *ompD* mRNA served as an Hfq binding site essential for the accelerated annealing of sRNAs to its coding sequence. The data suggest that the contributions of Hfq to the annealing of each sRNA to the complementary region in *ompD* mRNA were individually tuned depending on the structures of sRNAs and the structural contexts of their binding sites in mRNA. These results support the view of Hfq as a generic multifaceted binder of RNA molecules, the versatile roles of which in RNA metabolism are specified by the properties of interacting RNA molecules.

## MATERIALS AND METHODS

### RNA preparation

sRNAs and fragments of *ompD* mRNA were synthesized using T7 RNA polymerase (Milligan et al. 1987). The templates for the in vitro transcription of wt sRNAs and their mutants and fragments of *ompD* mRNA shorter than 140 nt were obtained by Taq polymerase extension of chemically synthesized overlapping oligodeoxyribonucleotides (oligo.pl) (Supplemental Table S3). The templates for the transcription of the longer fragments of *ompD* mRNA were obtained by PCR amplification from a pGEM T-Easy plasmid (Promega) containing a DNA sequence corresponding to nucleotides −103 to +210 of *ompD* mRNA from *Salmonella typhimurium*. To obtain templates for synthesis of mutated *ompD* mRNA fragments, the QuikChange Site-Directed Mutagenesis Kit (Stratagene) and specific primers (Supplemental Table S3) were used to introduce mutations into the *ompD* sequence in the pGEM T-Easy plasmid. After transcription the RNA molecules were purified using denaturing gel electrophoresis as previously described (Olejniczak 2011). RNAs were 5′-^32^P-labeled using T4 polynucleotide kinase or 3′ end labeled with RNA ligase, which was followed by phenol–chloroform extraction, gel purification, and ethanol precipitation.

Chemically synthesized oligoribonucleotides SdsR-18 (sequence: GCCUGCAUUAAUGCCAAC), MicC-12 (sequence: GUUAUAUGCCUU), *ompD*-19S (sequence: CCUGUUGGCAGCAGGCGUU), and *ompD*-18M (sequence: GCCGAGGUAUAUAACAAA) were kind gifts of Professor Ryszard Kierzek (Institute of Bioorganic Chemistry of the Polish Academy of Sciences). The oligoribonucleotides RybB-16 (sequence: GCCACUGCUUUUCUUU) and *ompD*-21R (sequence: UAAGUUAGUGGCAGUGGCAGU) were purchased from Metabion International AG.

### Hfq protein purification

The *Salmonella* Hfq protein with His_6_-tag on C-terminus was expressed from a pET15b vector (Novagen), in which the Hfq sequence was cloned via NcoI and BamHI restriction sites. This plasmid was used as a template for the preparation of Hfq R16A, Hfq Y25D, and Hfq K56A mutants using QuikChange Site-Directed Mutagenesis Kit (Stratagene) and specific primers (Supplemental Table S3). All constructs were verified by sequencing. Wt Hfq and its mutants were overexpressed and purified as described for *E. coli* Hfq (Małecka et al. 2015). The Hfq concentration was determined from absorption at 280 nm as previously described (Olejniczak 2011).

### Equilibrium binding assays

To monitor the equilibrium binding of ^32^P-labeled RNAs to the Hfq protein, a gel shift assay was used. Reactions were carried out in 1× binding buffer (Lease and Woodson 2004) supplemented with 2 mM MgCl_2_ (24 mM Tris–HCl pH 7.5, 50 mM NaCl, 50 mM NH_4_Cl, 50 mM KCl, 0.5 mM EDTA, 2 mM MgCl_2_, and 5% glycerol) at room temperature. Prior to use, RNAs were heated for 1 min at 90°C followed by cooling for 10 min at room temperature.

The equilibrium binding reactions were prepared by mixing 15 μL of 5′-^32^P-labeled RNA (0.02 nM concentration) with 15 μL of Hfq dilutions for 1 h at RT. Twenty microliters of each sample was loaded on a 6% native polyacrylamide gel in 1× THEM2 (66 mM HEPES, 34 mM Tris, 0.1 mM EDTA, 2 mM MgCl_2_) (Peng et al. 2014b). Gels were dried, exposed to phoshor screens, and visualized using Fujifilm phosphorimager (FLA 5000) with MultiGauge software. The fraction bound in individual complexes was calculated as a proportion of the total counts in each lane. When one complex of RNA with Hfq was formed, data were fit to the Michaelis–Menten binding isotherm. When two RNA complexes with Hfq were formed, the data were fit to a partition function assuming two unequal independent binding sites, and when three complexes were formed, the data were fit to a partition function assuming one specific and two equal unspecific sites (Soper et al. 2011).

### Kinetics of sRNA–mRNA annealing

The kinetics of RNA annealing was monitored by native mobility gel shift assays. RNAs were prepared as described above. All binding reactions were carried out at RT in 64 µL volume in 1× binding buffer. In the reactions, 1 nM ^32^P-labeled RNA was mixed with 25 nM RNA in the presence or absence of 3 nM Hfq hexamer. Native 6% polyacrylamide gels in 1× THEM2 were run continuously and aliquots were loaded onto the gel at specific time points (0.5–60 min). Controls were prepared in the same way as reactions, except that in the control reaction containing *ompD*-187 mRNA with Hfq the 2 nM concentration of *ompD*-187 was added to 1 nM ^32^P-labeled *ompD*-187 to keep the concentration of RNA the same as the concentration of Hfq to prevent RNA-Hfq complex retention in the wells. Controls were loaded before the first time point or after the last time point. After electrophoresis, gels were treated as described above. The fraction bound of individual complexes was calculated as a proportion of the total counts in each lane (RNA in complexes and free RNA). The fractions of sRNA–mRNA or sRNA–mRNA–Hfq complexes were plotted versus time (0.5–60 min). Observed association rate (*k*_obs_) values were calculated by fitting data to the single-exponential or double-exponential association equation, as described. For those reactions that were not complete at the last timepoint, the endpoint value of 70% was assumed in the data fitting.

### In vitro structure probing, footprinting, and boundary experiments

In vitro structure probing reactions were performed on 5′ end-labeled RNAs in a total volume of 10 µL. The RNA was denatured at 90°C for 1 min followed by incubation at room temperature for 10 min. Secondary structure probing with RNase T1 was performed in 12 mM Tris–HCl, pH 7.2, 48 mM NaCl, and 1.2 mM MgCl_2_ at RT for 10 min. RNase T2 digestion was carried out in 10 mM Tris pH 7, 100 mM KCl, and 10 mM MgCl_2_. RNase T2 reactions contained 1 µg of total yeast RNA (Ambion) and were incubated at RT for 15 min. To map nuclease S1 cleavage sites, RNA was incubated in 40 mM sodium acetate pH 4.5, 300 mM NaCl, and 2 mM ZnSO_4_ at RT for 10 min. RNase III digestions were performed in the same buffer as RNase T2 digestions, except for the addition of 1 mM DTT. Reactions were incubated at 37°C for 5 min. Reactions with RNase T1, T2, S1, and RNase III were quenched by addition of 10 µL of stop buffer (8 M urea and 20 mM EDTA). Formamide ladder was obtained by incubating ^32^P-labeled RNA in formamide (1:5 proportion) at 100°C for 1 h. Reaction was stopped by cooling on ice. To obtain RNase T1 ladder, ^32^P-labeled RNA was incubated in 50 mM sodium citrate (pH 4.3) and 7 M urea at 55°C for 10 min. Reaction was quenched by adding 10 µL of stop buffer. Samples were loaded on a 10% PAGE. Gels were frozen and exposed to phosphor screens overnight. Data were quantified using PhosphorImager.

To monitor changes in RNase accessibility of *ompD* mRNA structure upon sRNA binding, 5′ end-labeled *ompD*-187 or 5′ end-labeled *ompD*-93-187 were prepared as described above and then mixed with appropriate sRNA concentration in S1 reaction buffer. After 20 min of incubation, 1 µL of nuclease S1 (2U) was added to each reaction. After incubation at RT for 10 min, reactions were stopped and processed as described above.

To determine the location of Hfq binding sites within *ompD* mRNA leader, boundary experiments were performed. 5′ end-labeled or 3′ end-labeled *ompD*-187 molecules were prepared as described above and then subjected to RNase T1 or nuclease S1 cleavage in T1 or S1 reaction buffer, respectively. Reactions were incubated at RT for 10 min and stopped by addition of EDTA to the final concentration of 10 mM. Then, reactions were mixed with appropriate Hfq concentration. After 20 min of incubation at RT, 10 µL of 8 M urea was added to each tube, reaction products were resolved on 10% polyacrylamide gel in denaturing conditions, and analyzed as described above.

## SUPPLEMENTAL MATERIAL

Supplemental material is available for this article.

## Supplementary Material

Supplemental Material
