# Supplementary material for: Hfq assists small RNAs in binding to the coding sequence of ompD mRNA and in rearranging its structure
Source: RNA. 2016 Jul;22(7):979–94. doi: 10.1261/rna.055251.115 (PMC4911921; doi:10.1261/rna.055251.115)
Supplement: Supplemental Material [file supp_22_7_979__index.html]

Supplemental Material 

# Hfq assists small RNAs in binding to the coding sequence of *ompD* mRNA and in rearranging its structure

## Supplemental Material

**Files in this Data Supplement:**

- Supp Material.pdf
