# Supplementary material for: Hfq assists small RNAs in binding to the coding sequence of ompD mRNA and in rearranging its structure
Source: RNA. 2016 Jul;22(7):979–94. doi: 10.1261/rna.055251.115 (PMC4911921; doi:10.1261/rna.055251.115)
Supplement: Supplemental Material [file supp_055251.115_SuppMaterial.pdf]

SUPPLEMENTARY DATA

SUPPLEMENTARY TABLES

Supplementary Table 1. **The effect of mutations in the ARN motifs on the equilibrium binding of Hfq to *ompD*-187.**[a]

**^{32}P-RNA**	**K_d [nM]**	
	K_s	K_{ns}
***ompD*-187**	0.61 ± 0.17[b]	1.9 ± 0.58[b]
***ompD-187* ARN-1**	0.77 ± 0.097	3.0 ± 0.46
***ompD-187* ARN-2**	0.85 ± 0.003	2.2 ± 0.25
***ompD-187* ARN-3**	0.93 ± 0.24	2.9 ± 0.35
***ompD-187* ARN-5**	0.89 ±0.093	3.0 ± 0.46
***ompD-187* ARN-6**	0.82 ± 0.075	2.4 ± 0.29
***ompD-187* ARN-5+6**	0.81 ± 0.09	5.0 ± 1.5

Numbers are averages of at least three independent experiments. Data were fit to a partition function for one specific and two equal nonspecific binding sites. K_s – equilibrium dissociation constant for specific site binding, K_{ns} equilibrium dissociation constant for nonspecific site binding.

[a] – mutations in ARN motifs are shown on Suppl. Fig. 6

[b] – values from Table 1

Supplementary Table 2. **The effect of mutations in ARN motifs on the rates of sRNA annealing to *ompD*-187.**[a]

^{32}**P-RNA**	k_{obs} **[min^{-1}]**			
	**RybB**		**MicC**	
	-Hfq	+Hfq	-Hfq	+Hfq
***ompD*-187**	0.032 ± 0.005[b]	8.8 ± 0.7[b]	0.025 ± 0.0037[b]	6.9 ± 0.79 [b]
***ompD*-187 ARN-1**	0.022 ± 0.0057	7.2 ± 1.2	0.022 ± 0.0019	6.5 ± 0.96
***ompD*-187 ARN-2**	0.014 ± 0.0008	2.9 ± 0.9	0.024 ± 0.0048	3.1 ± 1.0
***ompD*-187 ARN-3**	0.022 ± 0.009	3.2 ± 1.1	0.019 ± 0.0058	2.1 ± 0.58
***ompD*-187 ARN-5**	0.037 ± 0.013	3.3 ± 0.91	0.011 ± 0.002	0.27 ± 0.05
***ompD*-187 ARN-6**	0.021 ± 0.0039	2.6 ± 0.86	0.012 ± 0.0029	1.1 ± 0.063
***ompD*-187 ARN-5+6**	0.012 ± 0.0023	3.2 ± 0.57	0.011 ± 0.0023	0.17 ± 0.031

Numbers are averages of at least three independent experiments.

[a] – mutations in the ARN motifs are shown on Suppl. Fig. 6

[b] – values from Table 2

Supplementary Table 3. **DNA oligonucleotides used in this study.**

**Name**	**Sequence in 5' to 3' direction**
ompD_-103_+210_F	GAACTTATGCCACTCCGTC
ompD_-103_+210_R	CTGATCGTTGATCTGCGTTTC
ompD-187_F	TTTTCTCGAGTTAATACGACTCACTATAGGCCATTGACAAACG
ompD-187_R	CGTGAACTTTACCGTACA
ompD-177_R	ACCGTACAGATCCAGTTTATTG
ompD_1-95_F	TAATACGACTCACTATAGCCATTGACAAACGCCTCGTTTAACAATGGTTG AGGAAACACGCTAAGA
ompD_1-95_R	GCCACTGCCACTAACTTAAGTTTCATTTTAATAATCCTTATAATTTTCTTA GCGTGTTTCCTCAAC
ompD_35-131_F	TAATACGACTCACTATAGGAAACACGCTAAGAAAATTATAAGGATTATT AAAATGAAACTTAAGTTAGT
ompD_35-131_R	GCATTTACAACGCCTGCTGCCAACAGGGAAGTCACTGCCACTGCCACTAA CTTAAGTTTCATTTT
ompD_93-187_F	TAATACGACTCACTATAGGCAGTGACTTCCCTGTTGGCAGCAGGCGTTGT AAATGCAGCCGAGG
ompD_93-187_R	CGTGAACTTTACCGTACAGATCCAGTTTATTGCCGTCTTTGTTATATACCT CGGCTGCATTTACAACG
ompD-131_R	GCATTTACAACGCCTGCTGCC
ompD-131 ARN-3_F	TAATACGACTCACTATAGGCCATTGACAAACGCCTCGTTTAACAATGGTT GAGGAAACACGCTTTGATAATTAT
ompD-131 ARN-3_R	GCATTTACAACGCCTGCTGCCAACAGGGAAGTCACTGCCACTGCCACTAA CTTAAGTTTCATTTTAATAATCCTTATAATTATC
ompD-131 SL2mut_R	GCATTTACAACGCCTGCTGCCAACAGGGAAGTCACTGCCACTGCCACTAA

	CTTAAGCATCATAAAAATAAGCCCTAGAATTATC
ompD-187 ΔSL2_F	TAATACGACTCACTATAGGCCATTGACAAACGCCTCGTTTAACAATGGTT GAGGAAAGCAGTGACTTCCCTGTTGGCAGCAGGCG
ompD-187 ΔSL2_R	CGTGAACTTTACCGTACAGATCCAGTTTATTGCCGTCTTTGTTATATACCT CGGCTGCATTTACAACGCCTGCTGCCAACAGGGAAG
ARN-1_F	GACAAACGCCTCGTTTCGCAATGGTTGAGG
ARN-1_R	CCTCAACCATTGCGAAACGAGGCGTTTGTC
ARN-2_F	CGTTTAACAATGGTTGTTGATACACGCTAAGAAAATTATAAGG
ARN-2_R	CCTTATAATTTTCTTAGCGTGTATCAACAACCATTGTTAAACG
ARN-3_F	GGTTGAGGAAACACGCTTTGATATTATAAGGATTATTAAAATG
ARN-3_R	CATTTTAATAATCCTTATAATATCAAAGCGTGTTTCCTCAACC
ARN-5_F	CGAGGTATATAACGAGGACGGCAATAAACTGG
ARN-5_R	CCAGTTTATTGCCGTCCTCGTTATATACCTCG
ARN-6_F	CAAAGACGGCCATTCACTGGATCTGTACGG
ARN-6_R	CCGTACAGATCCAGTGAATGGCCGTCTTTG
ARN-5+6_F	CGAGGTATATAACGAGGACGGCCATTCACTGG
ARN-5+6_R	CCAGTGAATGGCCGTCCTCGTTATATACCTCG
ompD-187_G94C_F	GTTAGTGGCAGTGCCAGTGACTTCCCTG
ompD-187_G94C_R	CAGGGAAGTCACTGGCACTGCCACTAAC
ompD-187_C113G_F	GTGACTTCCCTGTTGGGAGCAGGCGTTGTAAATGC
ompD-187_C113G_R	GCATTTACAACGCCTGCTCCCAACAGGGAAGTCAC
ompD-187_G139C_F	CGTTGTAAATGCAGCCGAGCTATATAACAAAGACGGC
ompD-187_G139C_R	GCCGTCTTTGTTATATAGCTCGGCTGCATTTACAACG
His-Hfq_ S.th_F	CCATGGCTAAGGGGCAATCTTTACAAG

His-Hfq_ S.th_R	TGGATCCTTAGTGATGGTGATGGTGATGTTCGGACTCTTCGCTG
Y25D_F	CCAGTTTCTATTGATTTGGTGAATGGTATT
Y25D_R	AATACCATTCACCAAATCAATAGAAACTGG
K56A_F	CAGCCAGATGGTTTATGCGCACGCGATTTCTACT
K56A_R	AGTAGAAATCGCGTGCGCATAAACCATCTGGCTG
R16A_F	TTCCTGAACGCATTGGCTCGGGAACGTGTTCCAG
R16A_R	CTGGAACACGTTCCCGAGCCAATGCGTTCAGGAA
RybB_F	TAATACGACTCACTATAGCCACTGCTTTTCTTTGATGTCCCCATTTTGTGG AGCCCATC
RybB_R	AAAAAACCCACCAACCTTGAACCGAAATGGCGGGGTTGATGGGCTCCAC AAAATGG
RybB_C2G_F	TAATACGACTCACTATAGGCACTGCTTTTCTTTGATGTCCCCATTTTGTGG AGCCCATC
SdsR_F	TAATACGACTCACTATAGCAAGGCGATTTAGCCTGCATTAATGCCAACTT TTAGCGCACGGCTCTC
SdsR_R	AAAAAGAGACCGAATACGATTCCTGTATTCGGTCCAGGGAAATGGCTCT TGGGAGAGAGCCGTGCGCTAAAAG
SdsR_G26C_F	TAATACGACTCACTATAGCAAGGCGATTTAGCCTGCATTAATCCCAACTT TTAGCGCACGGCTCTC
MicC_F	TAATACGACTCACTATAGTTATATGCCTTTATTGTCACATATTCATTTTGT CGCTGGGCCATTGCGTTAACC
MicC_R	AAAAAAAGCCCGAACATCCGTTCGGGCTTGTCAATTTATACGCTGGAAA GCAAAGGTTAACGCAATGGCCCAG
MicC_C9G_F	TAATACGACTCACTATAGTTATATGGCTTTATTGTCACATATTCATTTTGT CGCTGGGCCATTGCGTTAACC

SUPPLEMENTARY FIGURES

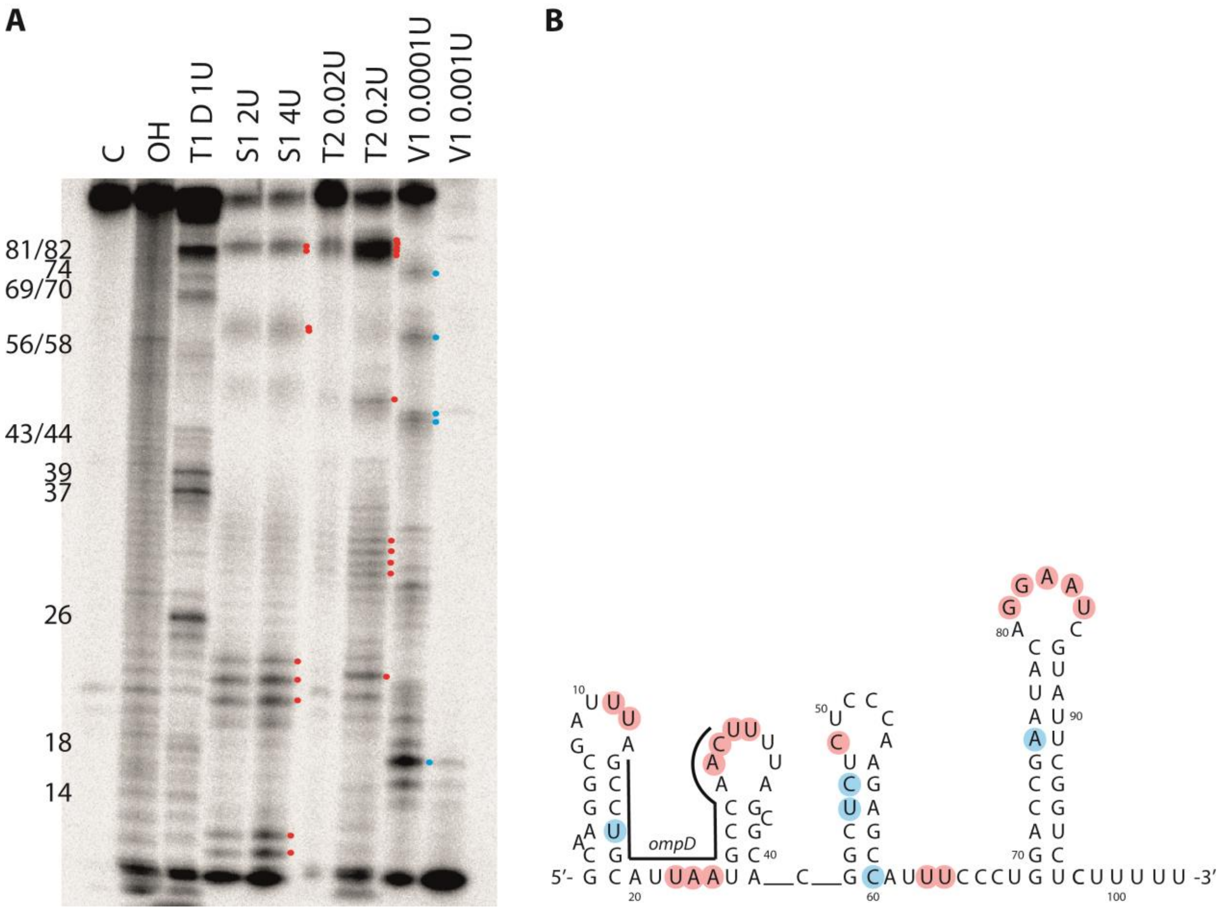

Suppl. Figure 1. **The secondary structure of SdsR sRNA.** (A) The structure probing of 5′-^{32}P-labeled SdsR using RNases indicated above the lanes. The untreated *ompD*-187 sample was resolved in lane marked C, formamide ladder in lane OH, and reaction with RNase T1 in denaturing conditions in lane T1 D. The positions of G-specific cleavages by RNase T1 are indicated on the left side of the gels. (B) The secondary structure model of SdsR sRNA proposed by *RNAstructure* program. Residues constrained as single-stranded are indicated with red circles, while double-stranded are indicated with blue circles. The region of SdsR involved in the pairing to *ompD* mRNA is marked with a line on the SdsR sRNA structure.

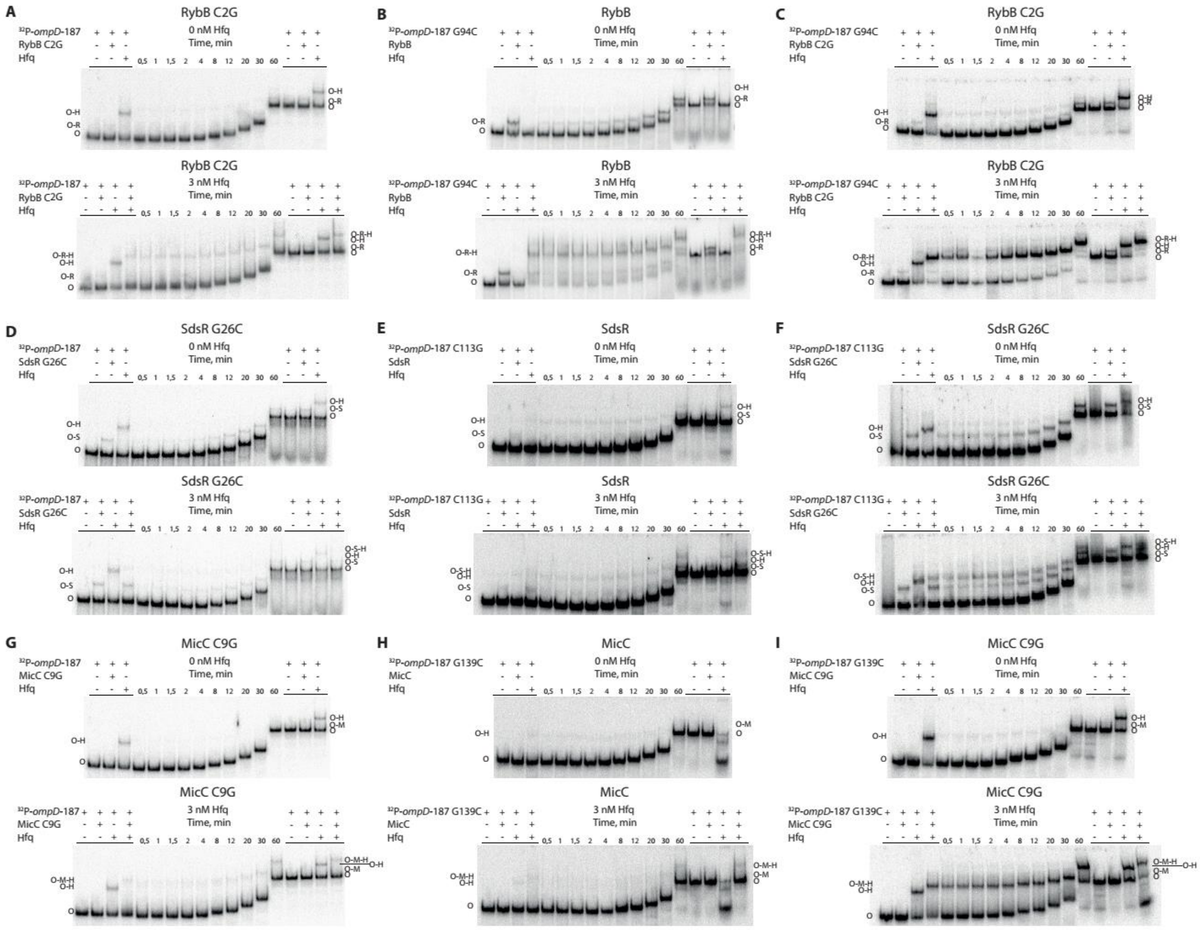

Suppl. Figure 2. **Compensatory mutations confirm the sites of annealing of sRNAs to *ompD* mRNA.** The native gel mobility shift assays were used to monitor the annealing of ^{32}P-labeled *ompD*-187 or its mutants at 1 nM concentration to sRNA molecules or their mutants at 25 nM concentration, in the absence or presence of 3 nM Hfq. The data show the annealing of (A) wt *ompD*-187 to RybB C2G, (B) *ompD*-187 G94C to wt RybB, (C) *ompD*-187 G94C to RybB C2G, (D) wt *ompD*-187 to SdsR G26C, (E) *ompD*-187 C113G to wt SdsR, (F) *ompD*-187 C113G to SdsR G26C, (G) wt *ompD*-187 to MicC C9G, (H) *ompD*-187 G139C to wt MicC, and (I) *ompD*-187 G139C to MicC C9G. Free RNAs and their complexes were marked as: O - free *ompD*-187; O-H - *ompD*-187-Hfq complex; O-R - *ompD*-187-RybB complex; O-R-H - *ompD*-187-RybB-Hfq ternary complex; O-S - *ompD*-187-SdsR complex; O-S-H - *ompD*-187-SdsR-Hfq ternary complex; O-M - *ompD*-187-MicC complex; O-M-H - *ompD*-187-MicC-Hfq ternary complex. The control reactions of ^{32}P-labeled *ompD*-187 binding to Hfq were supplemented with 2 nM cold mRNA to obtain the total concentration of mRNA equal to that of Hfq. The location of compensatory mutations in the complementary regions of interacting RNAs is shown on Fig. 1B.

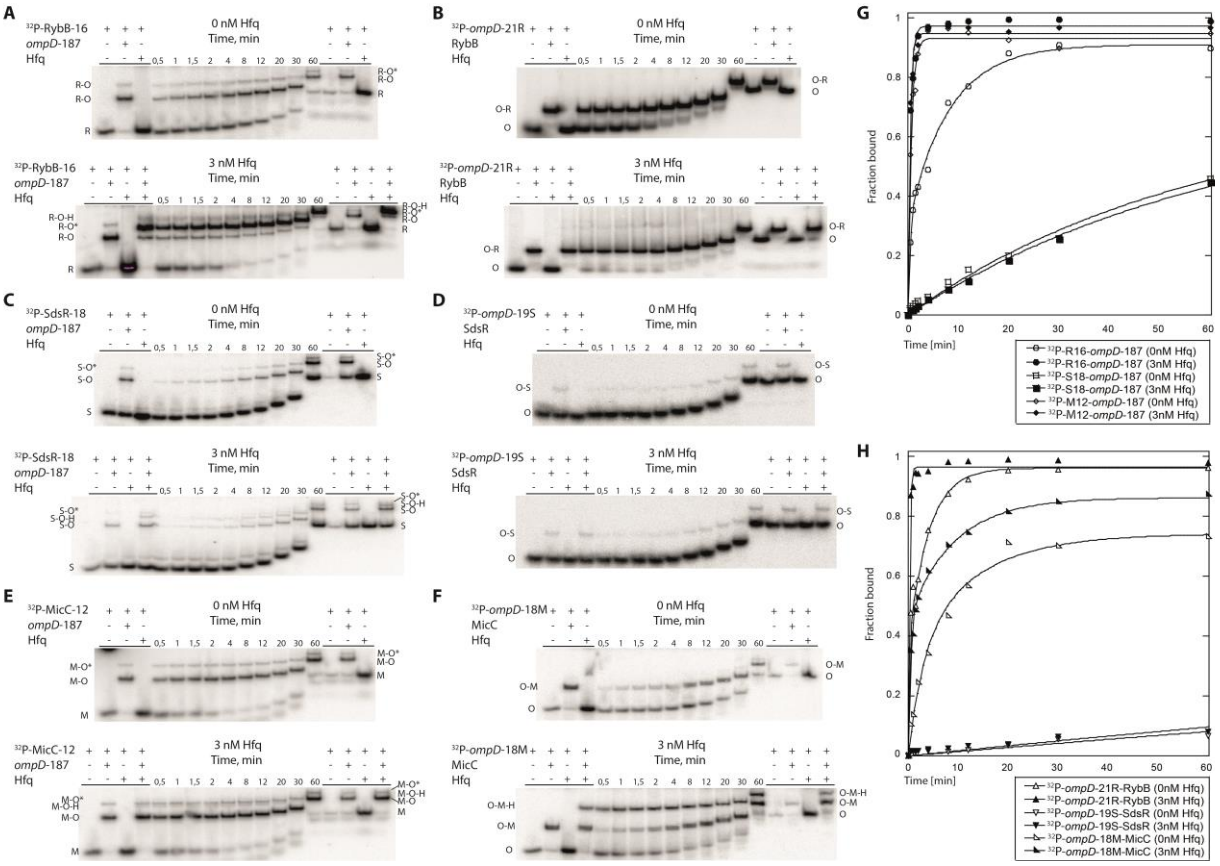

Suppl. Figure 3. **The rates of annealing of truncated sRNAs and *ompD* mRNA fragments.** The native gel mobility shift assays were used to monitor the annealing of (A) 1 nM ^{32}P-RybB-16 to 25 nM *ompD*-187, (B) 1 nM ^{32}P-*ompD*-21R to 25 nM RybB, (C) 1 nM ^{32}P-SdsR-18 to 25 nM *ompD*-187, (D) 1 nM ^{32}P-*ompD*-19S to 25 nM SdsR, (E) 1 nM ^{32}P-MicC-12 to 25 nM *ompD*-187, and (F) 1 nM ^{32}P-*ompD*-18M to 25 nM MicC in the absence or presence of 3 nM Hfq. Free RNAs and their complexes were labeled as: R - free RybB; R-O and R-O* - RybB-*ompD* complexes; R-H - RybB-Hfq complex; R-H-O - RybB-Hfq-*ompD* ternary complex; S - free SdsR; S-O and S-O* - SdsR-*ompD* complexes; S-H - SdsR-Hfq complex; S-H-O - SdsR-Hfq-*ompD* ternary complex; M - free MicC; M-O and M-O* - MicC-*ompD* complexes; M-H - MicC-Hfq complex; M-H-O - MicC-Hfq-*ompD* ternary complex. Controls of Hfq binding to *ompD*-187 were supplemented with 2 nM cold mRNA to obtain the total concentration of mRNA equal to that of Hfq. (G) Single exponential or double exponential (^{32}P-RybB-16 - *ompD*-187 in absence of Hfq) fits of data presented in (A), (C), and (E) versus time. (H) Single exponential or double exponential (^{32}P-*ompD*-21R - RybB in absence of Hfq and ^{32}P-*ompD*-18M - MicC in absence and presence of Hfq) fits of data presented in (B), (D), and (F) versus time.

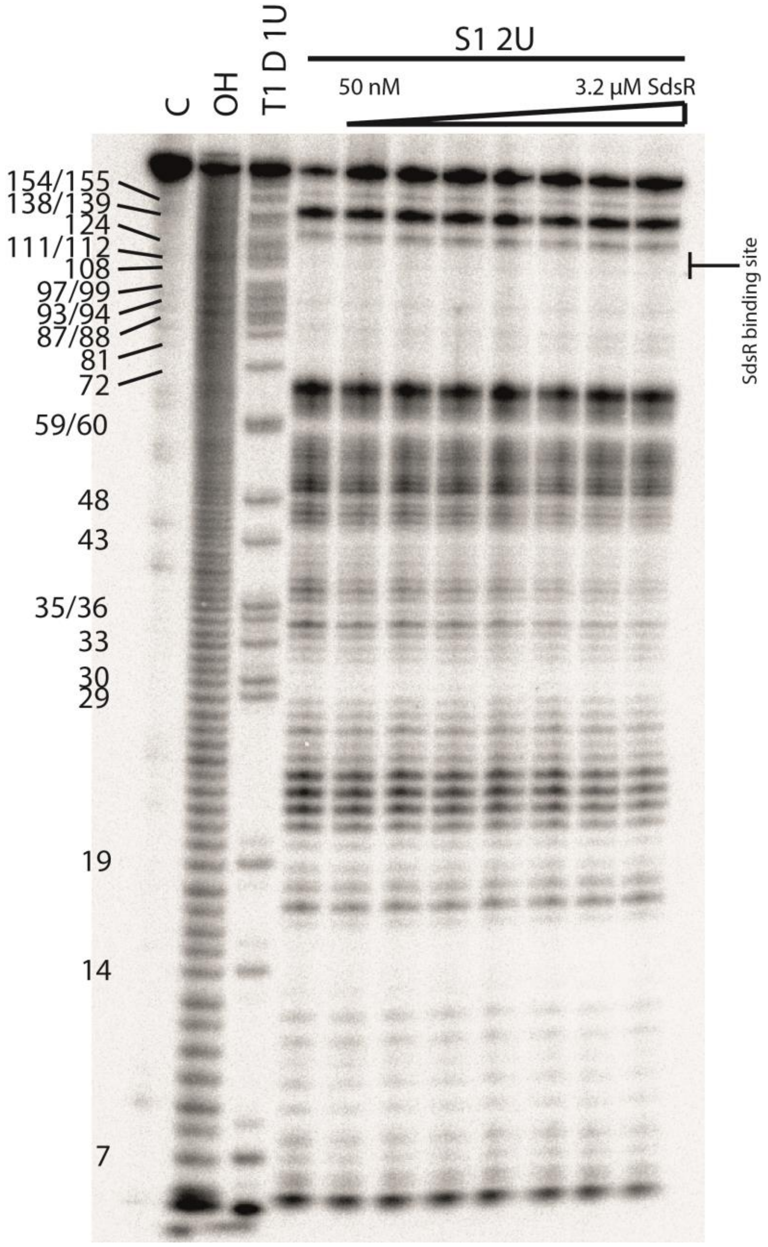

Suppl. Figure 4. **SdsR sRNA does not markedly affect the pattern of *ompD* mRNA structure probing.** The structure probing of 5′ ^{32}P-labeled *ompD*-187 with nuclease S1 at the increasing concentration of SdsR sRNA. The control untreated *ompD*-187 sample was resolved in lane C, formamide ladder in lane OH, and the products of reaction with RNase T1 in denaturing conditions in lane T1 D. The positions of G-specific cleavages by RNase T1 are indicated on the left side of the gel.

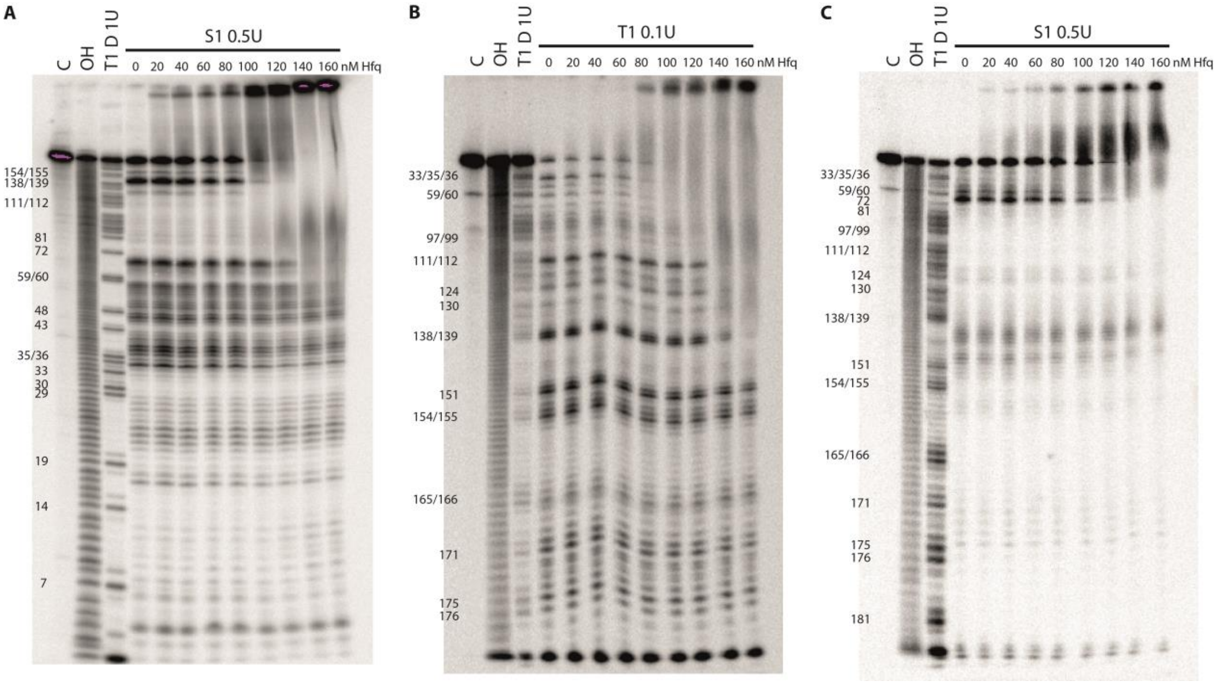

Suppl. Figure 5. **The boundary experiments identify the 5′-terminal and 3′-terminal regions, which contain Hfq binding sites.** The binding of Hfq to 5′-labeled *ompD*-187 degradation fragments obtained by partial digestion with nuclease S1 (A) and to 3′-labeled *ompD*-187 degradation fragments obtained by partial digestion with RNase T1 (B) or nuclease S1 (C). The control untreated *ompD*-187 samples were resolved in lanes marked C, formamide ladders in lanes OH, and reactions with RNase T1 in denaturing conditions in lanes marked T1 D. The positions of G-specific cleavages by RNase T1 are indicated on the left side of the gels.

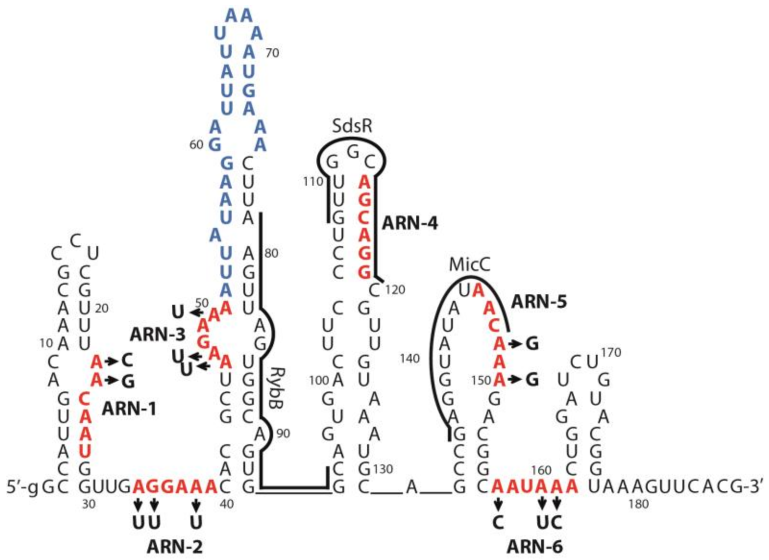

Suppl. Figure 6. **The nucleotide substitutions in the ARN motifs shown on the structure of *ompD*-187**. The mutants *ompD*-187 ARN-1, *ompD*-187 ARN-2, *ompD*-187 ARN-3, *ompD*-187 ARN-5, and *ompD*-187 ARN-6 contained mutations in single ARN motifs. The mutant *ompD*-187 ARN-5+6 contained mutations in both ARN-5 and ARN-6 motifs. The data describing the analysis of Hfq binding and sRNA annealing to these mutants are presented in Suppl. Tables 1 and 2.
